# Label-free anomaly detection in wastewater treatment plants via enhanced independent component analysis

**DOI:** 10.1038/s41598-026-51661-1

**Published:** 2026-05-06

**Authors:** K. Ramakrishna Kini, Fouzi Harrou, Muddu Madakyaru, Ying Sun

**Affiliations:** 1 Manipal Institute of Technology, Manipal Academy of Higher Education, Manipal, India; 2 Computer, Electrical and Mathematical Sciences and Engineering (CEMSE) Division, King Abdullah University of Science and Technology (KAUST), Thuwal, 23955-6900 Saudi Arabia

**Keywords:** Engineering, Mathematics and computing

## Abstract

Effective fault detection in wastewater treatment plants (WWTPs) is crucial for maintaining operational efficiency and preventing costly failures. This paper presents a semi-supervised fault detection framework that requires only fault-free data for training. The proposed method integrates Independent Component Analysis (ICA) for extracting statistically independent latent features from multivariate process data, combined with the Kolmogorov–Smirnov (KS) test for detecting distributional changes in residuals via sample-wise comparison. To ensure flexible and reliable thresholding, Kernel Density Estimation (KDE) is employed. The ICA–KS approach is evaluated using benchmark WWTP data across various fault types, including bias, drift, intermittent faults, freezing faults, and magnitudes, as well as simultaneous faults. Experimental results show that the method consistently outperforms traditional PCA- and ICA-based strategies, offering high accuracy and good sensitivity to weak and evolving faults.

## Introduction

Wastewater treatment plants (WWTPs) play a key role in protecting human and environmental health by treating wastewater to meet mandated discharge quality standards. Their reliable operation is crucial to preventing waterborne diseases, safeguarding aquatic ecosystems, and ensuring the availability of clean water^1–3^. WWTPs are inherently complex and dynamic, involving numerous interacting biological, chemical, and physical processes, which makes them susceptible to sensor faults, equipment failures, and process disturbances that can compromise treatment efficiency and lead to regulatory non-compliance^4,5^. Traditional monitoring methods often struggle with the nonlinear behavior, multivariate coupling, and stochastic fluctuations common in wastewater systems, which has driven extensive research into more advanced and data-driven anomaly detection techniques^6,7^. Therefore, robust monitoring and anomaly detection approaches are necessary to promptly identify abnormal conditions, support timely intervention, and maintain stable, compliant, and safe plant performance.

Monitoring and fault detection in WWTPs have been widely studied, and various approaches have been developed to detect sensor faults, process disturbances, and abnormal operating conditions^8,9^. Traditional univariate monitoring techniques, such as Shewhart, CUSUM, and EWMA control charts, are simple and efficient for detecting changes in individual variables^8,10^. However, WWTPs are highly multivariate systems where process variables are often correlated. In such cases, univariate techniques often fail to capture the underlying interactions among variables, resulting in delayed detection or increased false alarms. To address this, multivariate statistical process monitoring (MSPM) approaches have been introduced, with Principal Component Analysis (PCA) and Partial Least Squares (PLS) being the most widely used for dimensionality reduction and fault detection^11,12^. These methods have demonstrated improved performance by modeling variable correlations; however, they rely on assumptions of linearity and Gaussian data distributions, which may not hold in biological wastewater processes^4,13,14^.

Recent efforts in fault detection for WWTPs have advanced to tackle nonlinearity, incipient faults, and interpretability^5,7,15,16^. For instance, Xu et al.^13^ introduced a complex-valued slow independent component analysis (CSICA) to extract slowly varying, non-Gaussian features for early fault detection and root-cause diagnosis. Zhou et al.^12^ proposed an improved kernel extreme learning machine (KELM) approach enhanced by a mutation-based optimizer, utilizing kernel density estimation (KDE) to compute confidence intervals as fault thresholds, thereby achieving robust detection on BSM1 and real-world datasets. Navato et al.^17^ demonstrated the use of unsupervised K-means clustering on high-resolution UV–Vis spectrometry data to characterize influent modalities and isolate rare anomalies. Xu et al.^18^ developed a hybrid diagnostic model combining chaotic particle swarm optimization (CPSO), dynamic kernel principal component analysis (DKPCA), and Granger causality (GC) to address nonlinearity and temporal dependencies in WWTPs. Finally, Luca et al.^19^ employed Fisher discriminant analysis (FDA) to classify seven fault types affecting the dissolved oxygen (DO) sensor, achieving 87.5% accuracy while assessing the environmental and energetic impacts, notably greenhouse gas (GHG) emissions. These studies collectively reflect a shift toward hybrid, dynamic, and interpretable monitoring techniques tailored to the complexity of real-world WWTP environments.

Data-driven anomaly detection methods have been widely exploited in WWTPs due to their flexibility, scalability, and reduced reliance on detailed process models, which are often difficult to obtain or maintain for complex and dynamic biological systems. Compared to model-based methods, data-driven approaches can more effectively handle high-dimensional, nonlinear, and noisy data, making them suitable for real-time fault detection in WWTPs^4,7^. For instance, in^20^, a PCA-based sensor drift fault detection method with distribution adaptation (DAPCA) is proposed to address the multi-distributed and noisy nature of WWTP data. The approach combines temporal WaveCluster-based adaptive clustering with a robust PCA model and a smoothed composite monitoring index with adaptive thresholds, enabling improved detection of slow sensor drift faults. Experimental results on benchmark and real WWTP data show improved detection accuracy and fewer false alarms over conventional PCA methods. In^21^, a fuzzy principal component regression (FPCR) method is proposed for nonlinear modeling and adaptive monitoring in biological wastewater treatment plants (WWTPs). The approach integrates PCA for dimensionality reduction, adaptive fuzzy clustering for monitoring varying operating conditions, and a Takagi–Sugeno–Kang (TSK) fuzzy model to map PCA scores to output variables. FPCR effectively distinguishes between sustained faults and transient abnormalities while improving effluent quality prediction. In^2^, a multiscale principal component analysis (PCA) framework combined with Kantorovich distance (KD) monitoring is proposed for robust fault detection in noisy wastewater treatment data. By integrating wavelet-based filtering with nonparametric KD testing, the method enhances anomaly detection without requiring labeled data, outperforming conventional PCA-based techniques on the BSM1 benchmark across various sensor fault types and noise levels. In^22^, a Dynamic Multiblock Partial Least Squares (DMBPLS) method is proposed for quality-related monitoring of papermaking wastewater treatment processes. By augmenting input and output matrices to capture dynamic behavior, DMBPLS improves fault detection speed and interpretability and enables fault localization. Compared to traditional PLS, it achieves significantly higher detection rates, with improvements of 35.93% for bias faults and 12.5% for drift faults. In^23^, the authors propose a fault detection framework combining Uniform Manifold Approximation and Projection (UMAP) with Support Vector Data Description (SVDD) to handle high-dimensional, nonlinear, and non-Gaussian data in industrial wastewater treatment. The UMAP-SVDD approach achieves superior fault detection sensitivity and generalization compared to traditional linear models, ensuring improved adaptability and system integrity. In^24^, a semi-supervised anomaly detection framework is developed to detect faults in dissolved oxygen (DO) sensors and aeration valves. Five semi-supervised learning (SSL) models, Isolation Forest (IF), Local Outlier Factor (LOF), One-Class Support Vector Machine (OCSVM), Multilayer Perceptron Autoencoder (MLP-AE), and Convolutional Autoencoder (Conv-AE), are compared, with Conv-AE achieving the highest accuracy (up to 98.6%) across complete, concurrent, and complex faults. In^12^, an improved Kernel Extreme Learning Machine (KELM) combined with Kernel Density Estimation (KDE) is used to construct confidence intervals for effluent quality, enabling fault detection based on deviations from normal uncertainty bounds. The method demonstrates superior performance over CNN and LSTM models on both BSM1 and real wastewater treatment data. These recent advances highlight the growing effectiveness of data-driven techniques for fault detection in wastewater treatment; however, challenges remain in handling non-Gaussian behavior, capturing nonlinear interactions, and operating in the absence of labeled fault data.

To overcome the limitations of conventional monitoring approaches in handling non-Gaussian, nonlinear, and unlabeled process data, we propose a semi-supervised anomaly detection framework that integrates Independent Component Analysis (ICA) with the Kolmogorov–Smirnov (KS) test for robust distribution-based monitoring. The PCA strategy is widely used for dimensionality reduction based on second-order statistics and assumes that the underlying data are approximately Gaussian. As a result, its monitoring statistics and control limits are typically derived under Gaussian assumptions, which may not hold for real industrial processes. In contrast, ICA is employed to transform the multivariate sensor data into statistically independent components, allowing for better separation of latent, process-relevant signals from common-mode or correlated noise, a critical advantage in complex and dynamic systems such as WWTPs. This makes ICA particularly suitable for detecting subtle and incipient faults in complex process data. Additionally, a nonparametric KS test is applied to compare the empirical distribution of the ICA residuals against their reference distribution under normal operating conditions. These residuals, representing the modeling error or noise, tend to remain centered around zero when the system behaves normally, but deviate significantly from zero in the presence of abnormal conditions, thereby serving as sensitive indicators of potential faults. To further enhance detection reliability, we introduce a Kernel Density Estimation (KDE) step to construct smooth and adaptive thresholds from historical normal data. This allows the monitoring system to accommodate process variability while avoiding over-conservatism in detection. The entire scheme operates in a semi-supervised fashion, requiring only data from normal operating conditions for training, making it highly suitable for real-world WWTP applications where labeled fault samples are often unavailable or insufficient.

The proposed ICA–KS method is evaluated on the widely used Benchmark Simulation Model No. 1 (BSM1), under diverse and realistic sensor fault scenarios including bias, drift, intermittent faults, and freezing, each simulated at multiple magnitudes. The traditional ICA-based monitoring statistics, such as $I^{2}_{d}$, $I^{2}_{e}$, and SPE, are primarily based on second-order information (e.g., variance and energy of residuals). While they are effective in many cases, their sensitivity is limited when faults do not significantly alter the data’s mean or variance. In wastewater treatment processes, data are often non-Gaussian and complex, and faults may manifest as subtle changes in the underlying data distribution rather than large shifts in magnitude. In such cases, conventional statistics may fail to detect these anomalies. The Kolmogorov–Smirnov (KS) test is a non-parametric method that directly compares empirical distributions and is highly sensitive to distributional changes, including shifts, shape variations, and dispersion. By combining ICA with the KS test, the proposed method leverages ICA to extract independent and informative residuals, while KS detects changes in their distribution in a sample-by-sample manner using a moving window. This combination enables the detection of both strong and subtle faults that are often missed by traditional variance-based indicators, leading to improved detection accuracy and robustness in complex process environments. A comprehensive comparative analysis is conducted against established monitoring techniques, including PCA-$T^{2}$, PCA-Squared Prediction Error (SPE), PCA-KS, ICA-$I^{2}_{d}$, ICA-$I^{2}_{e}$, ICA-SPE, and ICA-KS, demonstrating the superior performance of the proposed method in terms of sensitivity and robustness to complex distributional shifts.

The remainder of this paper is organized as follows. Section "Methodological framework" introduces the methodological framework, including data preprocessing, ICA-based feature extraction, and the KS-based anomaly detection strategy. Section "Representation of the proposed ICA–KS-based fault detection strategy" presents the case study based on the BSM1 benchmark, describing the experimental setup and fault scenarios considered. Section "Results and discussion" reports and discusses the detection results and comparative performance analyses. Finally, Sect. "Conclusion" concludes the paper and outlines potential directions for future research.

## Methodological framework

The proposed monitoring approach consists of three main stages: (i) data preprocessing, (ii) feature extraction via Independent Component Analysis (ICA), and (iii) nonparametric anomaly detection based on the Kolmogorov–Smirnov (KS) test. The goal is to model the normal operating behavior of the WWTP using unlabeled historical data and subsequently detect departures from this behavior in real-time.

### Data preprocessing

In this study, the monitoring framework is built upon multivariate time-series measurements collected from the wastewater treatment process under normal operating conditions. The goal of preprocessing is to standardize the data so that all variables contribute fairly to the subsequent analysis and feature extraction steps. Let $\textbf{x}(t) = [x_1(t), x_2(t), \ldots , x_m(t)]^{\top } \in \mathbb {R}^{m}$ denote the vector of *m* synchronized process variables measured at time $t = 1, \ldots , T$ (e.g., dissolved oxygen, pH, ammonium, nitrate, flow rate, and temperature). Since these variables typically have different physical units and dynamic ranges, direct analysis may be biased toward signals with larger variances. To address this, each variable is standardized using the *z*-score transformation^20,22,24^:

1 $$\begin{aligned} \tilde{x}_i(t) = \frac{x_i(t) - \mu _i}{\sigma _i}, \qquad i = 1, \ldots , m, \end{aligned}$$

where $\mu _i$ and $\sigma _i$ are the mean and standard deviation of variable *i* estimated from a dataset corresponding exclusively to *normal (fault-free)* operating conditions. This ensures that the baseline model represents the inherent dynamics of healthy system behavior^2,23^. After normalization, the preprocessed dataset is arranged into the matrix

$$\tilde{\textbf{X}} = \begin{bmatrix} \tilde{\textbf{x}}(1)^{\top } \\ \tilde{\textbf{x}}(2)^{\top } \\ \vdots \\ \tilde{\textbf{x}}(N)^{\top } \end{bmatrix} \in \mathbb {R}^{N \times m},$$

where *N* denotes the number of normal samples used for model training.

### ICA-based feature extraction

Independent Component Analysis (ICA) is a powerful statistical technique for blind source separation, where the aim is to recover statistically independent latent signals from observed mixtures^25,26^. In the context of process monitoring, ICA has been widely adopted to uncover hidden factors driving variations in multivariate process data, enabling the detection of subtle abnormalities or developing faults that may not be directly visible in raw sensor measurements^27,28^. ICA assumes that the observed data vector $\tilde{\textbf{x}}(t)$ is generated by a linear combination of statistically independent source signals^25^:

2 $$\begin{aligned} \tilde{\textbf{x}}(t) = \textbf{A} \, \textbf{s}(t), \end{aligned}$$

where $\textbf{s}(t)$ denotes the independent components (ICs) and $\textbf{A}$ is the mixing matrix representing how latent process sources influence the measured variables.

A crucial preprocessing step in ICA is *whitening*, which removes second-order correlations between variables and simplifies the separation problem ?^26^. The whitening operation can be expressed as:

3 $$\begin{aligned} \tilde{\textbf{x}}(t) = \textbf{Q}\textbf{Z}_{c} = \textbf{Q}\textbf{A}\hat{\textbf{s}}(t) = \textbf{B}\hat{\textbf{s}}(t), \end{aligned}$$

where $\textbf{Q} = \boldsymbol{\Lambda }^{-1/2}\textbf{B}^{\top }$, $\boldsymbol{\Lambda }$ denotes the diagonal matrix of eigenvalues, and $\textbf{B}$ is the matrix of eigenvectors computed from the covariance matrix of the centered data $\textbf{Z}_{c}$.

The objective of ICA is to estimate a demixing matrix $\textbf{W}$ such that:

4 $$\begin{aligned} \hat{\textbf{s}}(t) = \textbf{W} \, \tilde{\textbf{x}}(t), \end{aligned}$$

thereby recovering statistically independent features that characterize the intrinsic behavior of the system. To achieve this separation, ICA maximizes statistical *non-Gaussianity*, commonly quantified using *negentropy*^29^:

5 $$\begin{aligned} J(s_i) = H(s_i^{\text {gaussian}}) - H(s_i), \end{aligned}$$

where $H(\cdot )$ denotes differential entropy. Since mixtures of independent signals tend to become more Gaussian according to the Central Limit Theorem, maximizing non-Gaussianity enables effective separation of independent sources^26^. Among ICA algorithms, FastICA provides an efficient fixed-point solution and has been extensively applied in industrial process monitoring due to its numerical stability and computational efficiency^27^. The update rule for each demixing vector is given by:

6 $$\begin{aligned} \textbf{w}_k^{\text {new}} = \mathbb {E}\{\tilde{\textbf{x}}\,g(\textbf{w}_k^{\top }\tilde{\textbf{x}})\} - \mathbb {E}\{g'(\textbf{w}_k^{\top }\tilde{\textbf{x}})\}\textbf{w}_k, \end{aligned}$$

where $g(\cdot )$ is a nonlinear contrast function (e.g., $\tanh (\cdot )$). After convergence, the matrix of independent components is obtained as:

7 $$\begin{aligned} \hat{\textbf{S}} = \tilde{\textbf{X}}\,\textbf{W}^{\top }. \end{aligned}$$

Once the ICs are extracted, a key advantage of ICA over PCA is its ability to handle non-Gaussian data^26,27^. While PCA extracts directions of maximum variance under the assumption of approximate Gaussianity, many WWTP variables exhibit nonlinear behavior, biological variability, and diurnal fluctuations, leading to strongly non-Gaussian distributions. As a result, PCA may mix fault-related information across several components, reducing its sensitivity to weak or incipient faults. ICA, by explicitly maximizing independence and non-Gaussianity, can isolate latent patterns more effectively, making it particularly suitable for detecting subtle process abnormalities and early-stage faults in wastewater treatment monitoring.

### Monitoring statistics based on ICA

Once the semi-supervised ICA model is trained using normal operating data, it can be employed for fault detection by monitoring deviations in newly observed data. Following established ICA-based process monitoring frameworks^30,31^, three complementary monitoring statistics are adopted: the squared distance in the retained independent component (IC) subspace ($I^{2}_{d}$), the squared distance in the residual IC subspace ($I^{2}_{e}$), and the Squared Prediction Error (SPE) in the original variable space.

The statistic $I^{2}_{d}$ captures abnormal variations along the dominant IC directions retained for monitoring, whereas $I^{2}_{e}$ reflects deviations in the discarded IC subspace, which may contain fault-related information. The SPE measures reconstruction errors between the original observations and their ICA-based estimates, thereby capturing residual variations not explained by the model. These statistics are defined as follows^32^:

8 $$\begin{aligned} I^{2}_{d}&= \textbf{Z}^{\top } \textbf{W}_{m}^{\top } \textbf{W}_{m} \textbf{Z}, \end{aligned}$$

9 $$\begin{aligned} I^{2}_{e}&= \textbf{Z}^{\top } \textbf{W}_{d-m}^{\top } \textbf{W}_{d-m} \textbf{Z}, \end{aligned}$$

10 $$\begin{aligned} \text {SPE}&= \textbf{e}^{\top } \textbf{e}, \end{aligned}$$

where the reconstruction error $\textbf{e}$ is given by

11 $$\begin{aligned} \textbf{e}&= \textbf{X}(i) - \hat{\textbf{X}}, \end{aligned}$$

12 $$\begin{aligned} \hat{\textbf{X}}&= \textbf{Q}^{-1} \textbf{B}_{m} \textbf{W}_{m} \textbf{Z}. \end{aligned}$$

Here, $\textbf{W}_{m} \in \mathbb {R}^{m \times d}$ contains the loading vectors of the retained ICs, while $\textbf{W}_{d-m} \in \mathbb {R}^{(d-m) \times d}$ corresponds to the discarded ICs. By jointly monitoring these statistics, faults can be effectively detected in both the dominant IC space and the residual subspaces, improving sensitivity to diverse anomaly types.

The residual contribution analysis has been incorporated to enable fault isolation in addition to fault detection. Once a fault is detected, contribution plots are used to identify the variables responsible for the abnormal behavior. These plots quantify the contribution of each process variable to the residuals, allowing clear identification of the root cause. The residuals are computed using the Eq. 11. The contribution plots are then represented as bar graphs, where each bar corresponds to the contribution of a variable at a given sample. Variables with significantly larger contributions indicate the likely source of the fault.

### Kolmogorov–Smirnov-based fault indicator

In statistical process monitoring, fault detection often relies on measuring the degree of similarity or dissimilarity between data distributions corresponding to normal and current operating conditions^33^. Distribution-based methods are particularly attractive for anomaly detection, as faults typically manifest as changes in the underlying data distribution rather than simple shifts in mean or variance. In this work, a nonparametric fault indicator based on the Kolmogorov–Smirnov (KS) test is employed to quantify distributional deviations between reference (normal) data and newly observed data.

The KS test compares the empirical cumulative distribution functions (eCDFs) of two samples and measures the maximum absolute distance between them^34^. Owing to its nonparametric nature, the KS test does not rely on any specific distributional assumptions, making it especially suitable for wastewater treatment process data, which are often nonlinear and non-Gaussian. In contrast to parametric tests that assume Gaussianity, the KS test remains valid for arbitrary distributions and is therefore robust to unknown or time-varying data characteristics^35^. Two variants of the KS test exist: the one-sample test and the two-sample test. The two-sample KS test is adopted in this study, as it directly compares two independent datasets–namely, the residuals obtained under normal operating conditions and those obtained during online monitoring. The presence of a fault is expected to induce a noticeable change in the eCDF of the monitoring residuals, which can be effectively captured by the two-sample KS statistic.

Let $\{y_{a1}, y_{a2}, \ldots , y_{a n_a}\}$ and $\{y_{b1}, y_{b2}, \ldots , y_{b n_b}\}$ denote two independent samples with eCDFs $A_{n_a}(y)$ and $B_{n_b}(y)$, respectively. The two-sample KS statistic is defined as^36^:

13 $$\begin{aligned} D_{n} = \sup _{y} \left| A_{n_a}(y) - B_{n_b}(y) \right| . \end{aligned}$$

Under the null hypothesis that both samples are drawn from the same distribution, the asymptotic distribution of the KS statistic is given by:

14 $$\begin{aligned} Q(\lambda ) = 2 \sum _{i=1}^{\infty } (-1)^{i-1} \exp (-2 i^2 \lambda ^2), \end{aligned}$$

where

15 $$\begin{aligned} \lambda = \sqrt{\frac{n_a n_b}{n_a + n_b}} \, D_{n}. \end{aligned}$$

A large KS statistic indicates a significant discrepancy between the two distributions, signaling abnormal behavior. For fault detection, the computed KS statistic is compared against a predefined threshold corresponding to a chosen significance level. Exceeding this threshold leads to the declaration of a fault, making the KS test an effective and flexible indicator for distributional changes in process monitoring applications.

## Representation of the proposed ICA–KS-based fault detection strategy

This study proposes a semi-supervised strategy for sensor fault detection in wastewater treatment plants (WWTPs). The core idea is to integrate Independent Component Analysis (ICA) for multivariate feature extraction with the Kolmogorov–Smirnov (KS) nonparametric test to monitor deviations in sensor behavior. The KS test evaluates the distributional difference between model residuals under normal and current conditions, enabling robust detection without assuming any specific data distribution.

ICA is used to extract statistically independent components from normalized process data, preserving latent non-Gaussian structures. The model generates residuals that reflect unexplained variations in the system. These residuals, typically centered around zero under normal conditions, exhibit significant deviation in the presence of faults, making them a valuable indicator for anomaly detection. The KS test quantifies distributional changes in these residuals across a sliding window, allowing sensitive and adaptable monitoring of both abrupt and incipient faults.

**Fig. 1:**
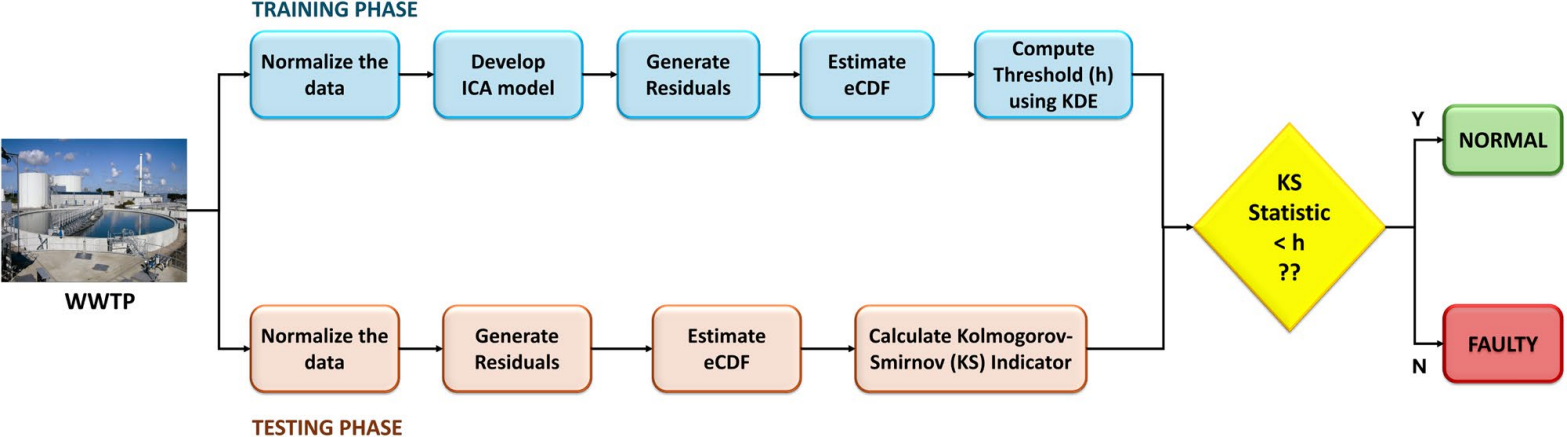
The proposed ICA–KS-based fault detection strategy.

Figure 1 illustrates the overall framework, comprising two main phases: training and testing.

### Training phase

The training phase begins with standardization of the multivariate time-series data to zero mean and unit variance. ICA is then applied to extract dominant independent components, resulting in the construction of the ICA model. The model is used to reconstruct the input signal, and residuals $\textbf{E}$ are computed as:

16 $$\begin{aligned} \textbf{E} = \textbf{X} - \hat{\textbf{X}} = \textbf{Q}^{-1} \textbf{B}_{m} \textbf{W}_{m} \textbf{Z}, \end{aligned}$$

where $\textbf{W}_m$ and $\textbf{B}_m$ represent the retained rows of the ICA demixing and whitening matrices, and $\textbf{Z}$ denotes the whitened input. These residuals form the baseline for reference behavior. A detection threshold is then established using Kernel Density Estimation (KDE), capturing the upper bound of residual variability under fault-free conditions.

### Testing phase

In the testing phase, incoming process data are normalized and projected through the reference ICA model to generate residuals $\textbf{F}$ using Eq.  16. Empirical cumulative distribution functions (eCDFs) of both training ($\textbf{E}$) and testing ($\textbf{F}$) residuals are calculated, denoted as $\textbf{L}$ and $\textbf{P}$, respectively. The KS statistic is computed in a moving window framework to detect changes in the residual distribution over time.

Specifically, the following steps are carried out:

1. Divide $\textbf{L}$ into overlapping windows $L_1, L_2, \ldots , L_n$ of length *j* using a sliding window of size *h*.
2. Similarly, partition $\textbf{P}$ into overlapping windows $P_1, P_2, \ldots , P_n$ of size *j*.
3. For each pair $(L_i, P_i)$, compute the KS statistic:
  $$D_i = \sup _y \left| A_{L_i}(y) - B_{P_i}(y) \right| ,$$
  where $A_{L_i}$ and $B_{P_i}$ are the eCDFs of the two samples.
4. A small $D_i$ implies similarity (fault-free), while a large $D_i$ suggests significant distributional change (potential fault).

Finally, the decision rule for fault detection is given as:

17 $$\begin{aligned} \delta = {\left\{ \begin{array}{ll} \text {Fault-free}, & \text {if } \text {KS} < \text {Threshold}; \\ \text {Faulty}, & \text {if } \text {KS} \ge \text {Threshold}. \end{array}\right. } \end{aligned}$$

This approach enables sensitive detection of diverse fault types, including bias, drift, intermittent failures, and freezing, with varying magnitudes. The use of ICA ensures that complex, non-Gaussian process variations are effectively captured, while the KS test provides a distribution-agnostic detection mechanism suitable for real-world WWTP monitoring environments.

The process is considered to operate under normal conditions when the Kolmogorov–Smirnov (KS) statistic remains below a predefined reference threshold. A fault is declared whenever the KS value exceeds this threshold, indicating a significant distributional shift in the residuals. In earlier studies, the KS threshold was often fixed using conventional significance levels (e.g., 0.01 or 0.05) without a principled criterion tailored to the underlying data distribution. However, assigning a static threshold without considering the empirical characteristics of the monitored process may lead to suboptimal fault detection performance and inconsistent false alarm behavior across different applications. To address this limitation, a data-driven threshold selection strategy based on Kernel Density Estimation (KDE) is adopted in this work. KDE provides a flexible and nonparametric estimate of the probability density function of the KS statistic under normal operating conditions, without assuming any specific parametric form. This is particularly advantageous for wastewater treatment processes, where the monitored data and resulting statistics often exhibit non-Gaussian and skewed distributions due to nonlinear dynamics and external disturbances.

Mathematically, the KDE of a set of KS statistics $\{z_1, z_2, \ldots , z_d\}$ obtained under fault-free conditions is given by^37^:

18 $$\begin{aligned} \hat{f}(z) = \frac{1}{d h} \sum _{i=1}^{d} K \left( \frac{z - z_i}{h} \right) , \end{aligned}$$

where $\hat{f}(z)$ denotes the estimated density function, $K(\cdot )$ is the kernel function (typically Gaussian), and *h* is the bandwidth parameter controlling the smoothness of the estimate.

Once the density function is obtained, the detection threshold is selected as a high percentile (e.g., 95% or 99%) of the estimated distribution. This ensures that, under normal operating conditions, only a small proportion of observations exceed the threshold, thereby controlling the false alarm rate while maintaining high sensitivity to abnormal behavior.

#### Computational complexity and real-time feasibility

The proposed ICA–KS-based fault detection framework is designed to be computationally efficient, making it suitable for real-time monitoring in WWTPs. After the ICA model is trained offline using historical fault-free data, the online phase of the algorithm involves only lightweight operations, specifically, the computation of residuals and the application of the KS test in a sliding window configuration. For each new observation $\textbf{x}(t) \in \mathbb {R}^m$, the residual is calculated as $\textbf{e}(t) = \textbf{x}(t) - \hat{\textbf{x}}(t) = \textbf{x}(t) - \textbf{Q}^{-1} \textbf{B}_m \textbf{W}_m \textbf{z}(t)$, where $\textbf{z}(t)$ is the whitened data vector. Since the ICA parameters $\textbf{Q}$, $\textbf{B}_m$, and $\textbf{W}_m$ are precomputed during training, the residual computation involves only a series of matrix-vector multiplications. This operation has a computational complexity of *O*(*md*), where *m* is the number of process variables and *d* is the number of retained independent components (ICs).

The KS test is applied to detect distributional shifts by comparing the empirical cumulative distribution functions (eCDFs) of the residuals from normal and current operating conditions over a fixed-size sliding window. For a window size *w*, the two-sample KS test requires sorting both samples ($O(w \log w)$), followed by a linear scan to compute the maximum absolute difference between the eCDFs (*O*(*w*)). Therefore, the overall computational complexity of the KS test is $O(w \log w)$ per time step. Combining both components, the total per-sample online complexity of the proposed monitoring strategy is $O(md + w \log w)$. Since both *d* and *w* are typically small and fixed, the method ensures rapid computation and scalability in practice. Additionally, the approach does not require labeled fault data or model re-training, further reducing its deployment burden. This efficiency, along with the robustness of the ICA–KS method in detecting non-Gaussian faults, makes it particularly suitable for real-time fault detection in complex industrial environments like WWTPs.

## Results and discussion

This section describes the WWTP data and evaluates the detection performance of the proposed MSPCA-KD strategy for identifying various sensor faults under noisy conditions. To quantify performance, four statistical metrics are used: fault detection rate (FDR), false alarm rate (FAR), precision, and F1-score. More details about these statistical indices can be found in^38^.

**Fig. 2:**
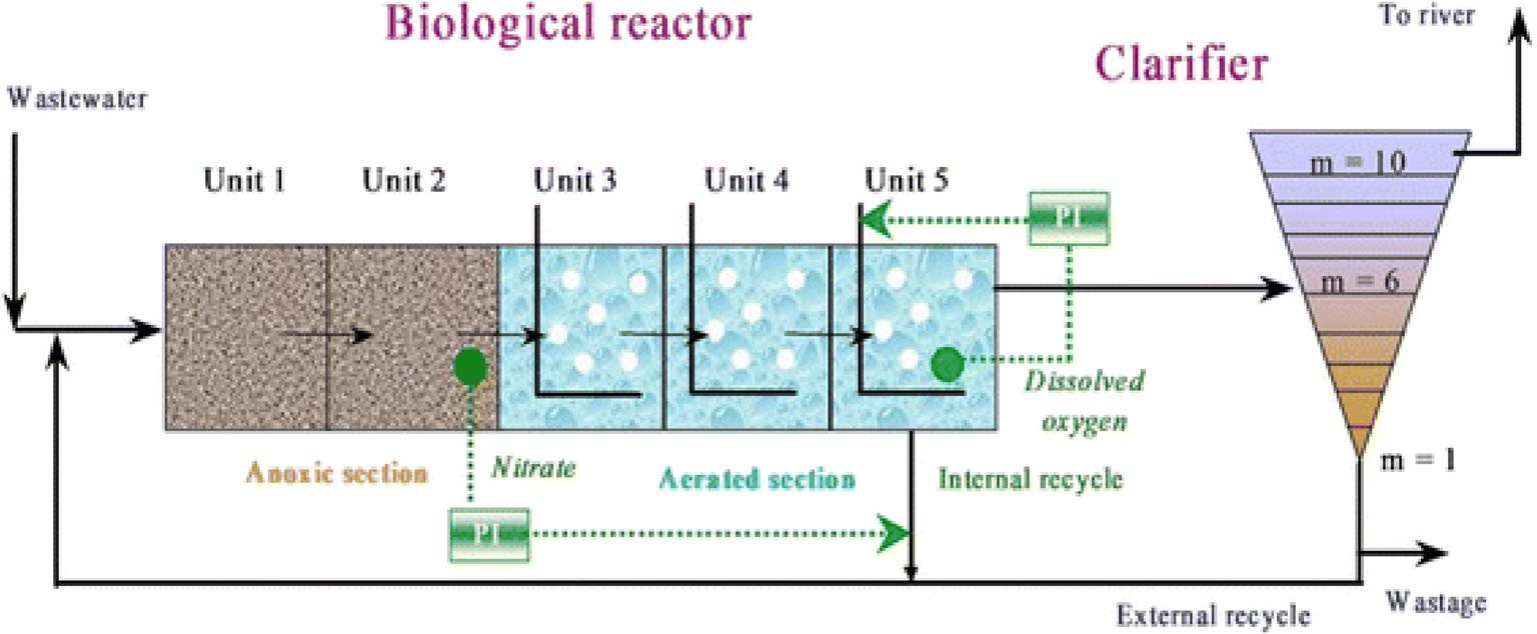
Schematic diagram of the BSM1 wastewater treatment process including anoxic and aerobic tanks, internal and external recirculation, and clarifier.

### BSM1 dataset and exploratory data analysis

The Benchmark Simulation Model No. 1 (BSM1) is a standardized and widely used simulation framework for evaluating control, monitoring, and optimization strategies in municipal wastewater treatment^39^. It represents a conventional activated sludge process, including biological reactions, sludge handling, and dynamic influent patterns, offering a realistic benchmark for comparative studies.

The BSM1 treatment line comprises a five-compartment biological reactor followed by a secondary clarifier. The biological reactor is designed to achieve effective nitrogen removal and includes two anoxic tanks (Units 1 and 2) followed by three aerobic tanks (Units 3 to 5). The anoxic zones primarily facilitate denitrification, while the aerobic sections support both organic matter degradation and nitrification processes. The hydraulic arrangement reflects practical process configurations observed in full-scale wastewater treatment plants. The hydraulic and biological dynamics are supported by multiple flow streams. The influent enters Unit 1 (first anoxic tank), initiating the biological treatment process. Nitrate-rich mixed liquor from the last aerobic tank (Unit 5) is recycled internally to the anoxic zone to support denitrification. In parallel, an external recycle of settled sludge from the clarifier returns to the head of the reactor, maintaining sufficient biomass. Excess sludge (WAS) is removed from the system at the end of the aerobic section for solids management. The layout of BSM1 follows a left-to-right horizontal configuration, clearly showing the progression of flow and interactions between compartments. Figure 2 illustrates this configuration, including the five biological units, internal and external recirculation loops, and the clarifier that separates treated effluent and sludge.

The influent data of the Benchmark Simulation Model No. 1 (BSM1) under dry weather conditions comprises eight key variables that describe the organic and nitrogen content of wastewater, as well as the flow rate. Table 1 lists the symbols, definitions, and units.

**Table 1:** Considered influent variables in BSM1 simulation.

Symbol	Definition	Unit
$S_S$	Readily biodegradable substrate	g COD m$^{-3}$
$X_I$	Particulate inert organic matter	g COD m$^{-3}$
$X_S$	Slowly biodegradable substrate	g COD m$^{-3}$
$X_{B,H}$	Active heterotrophic biomass	g COD m$^{-3}$
$S_{NH}$	Ammonium + ammonia nitrogen	g N m$^{-3}$
$S_{ND}$	Soluble biodegradable organic nitrogen	g N m$^{-3}$
$X_{ND}$	Particulate biodegradable organic nitrogen	g N m$^{-3}$
$Q_i$	Influent flow to the anoxic section	m$^3$ d$^{-1}$

**Fig. 3:**
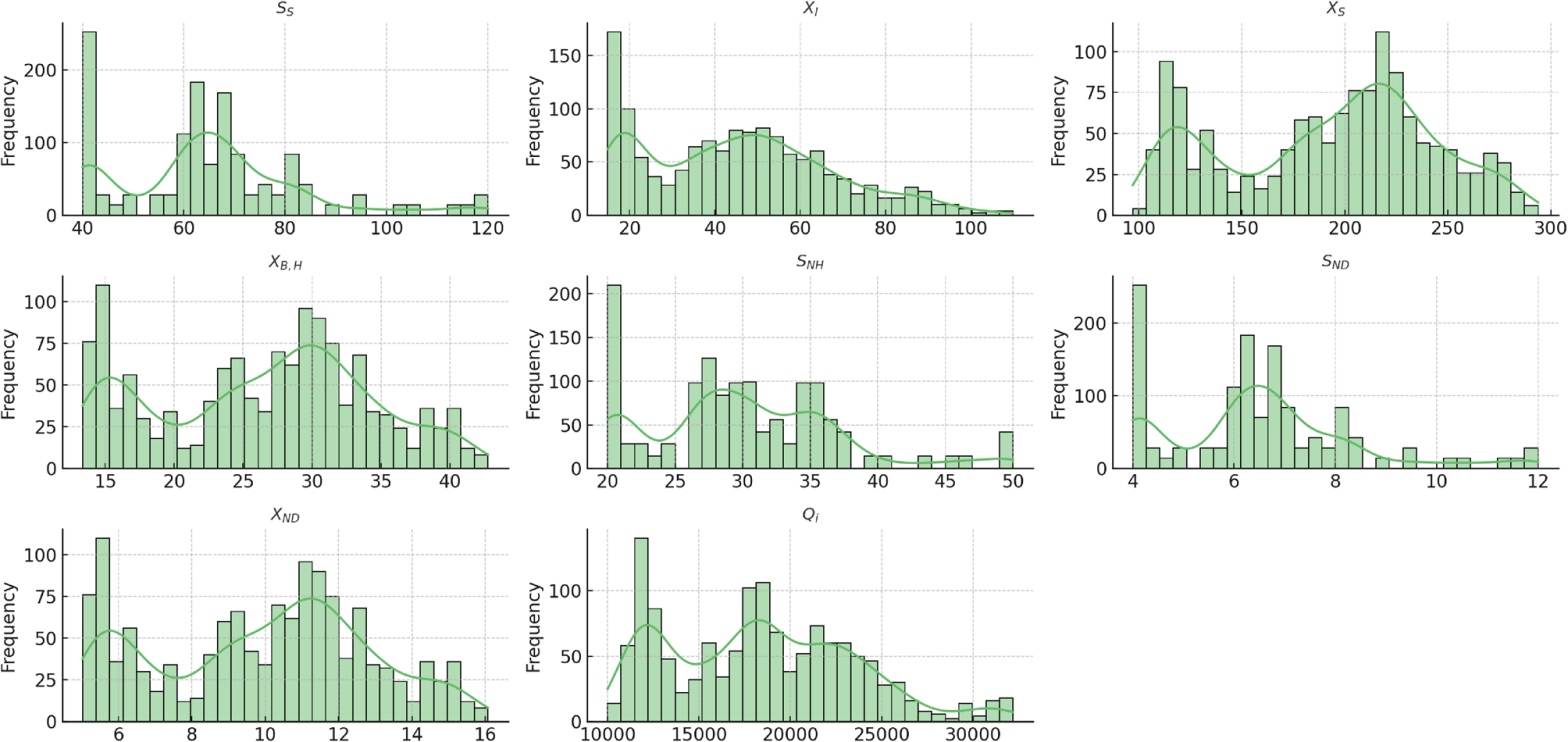
Histograms of BSM1 influent variables with KDE overlays (dry weather).

To understand the statistical behavior of each variable, Fig.  3 presents histograms with kernel density estimation (KDE) overlays for the eight influent variables.

The histograms reveal several key distributional characteristics of the influent variables (Fig.  3). Variables such as $S_S$, $X_S$, and $Q_i$ exhibit moderate right-skewness, indicating occasional higher loading events. In contrast, the nitrogen-related fractions, namely $S_{NH}$, $S_{ND}$, and $X_{ND}$, display more centralized and compact distributions, reflecting relatively stable nitrogen content under dry weather conditions. Additionally, some variables, particularly $X_{B,H}$ and $X_I$, show indications of multimodal behavior, which may suggest variability in influent composition or shifts in upstream wastewater characteristics.

Normality was assessed using the Shapiro–Wilk test, along with skewness and kurtosis indicators. The results are reported in Table 2. The results indicate that all p-values from the Shapiro–Wilk test are below 0.05, confirming statistically significant deviations from normality across all influent variables. The skewness values point to mild distributional asymmetry, particularly for $S_S$, $S_{NH}$, and $Q_i$, which display noticeable right-skewed behavior. Meanwhile, the kurtosis values lie between *platykurtic* (i.e., flatter than a normal distribution, as observed in $X_S$) and *mesokurtic* (i.e., similar to a normal distribution), suggesting that the variables generally do not exhibit heavy tails or a high prevalence of extreme outliers. These non-Gaussian characteristics in the data support the adoption of Independent Component Analysis (ICA), which is specifically designed to extract statistically independent and potentially non-Gaussian latent features, thereby enhancing the sensitivity of anomaly detection.

**Table 2:** Normality test results for influent variables (Shapiro–Wilk, skewness, kurtosis).

Variable	Shapiro–Wilk p-value	Skewness	Kurtosis
$S_S$	0.0000	0.796	0.855
$X_I$	0.0000	0.434	-0.470
$X_S$	0.0000	-0.217	-0.990
$X_{B,H}$	0.0000	-0.088	-0.967
$S_{NH}$	0.0000	0.643	0.417
$S_{ND}$	0.0000	0.796	0.855
$X_{ND}$	0.0000	-0.088	-0.967
$Q_i$	0.0000	0.401	-0.427

**Fig. 4:**
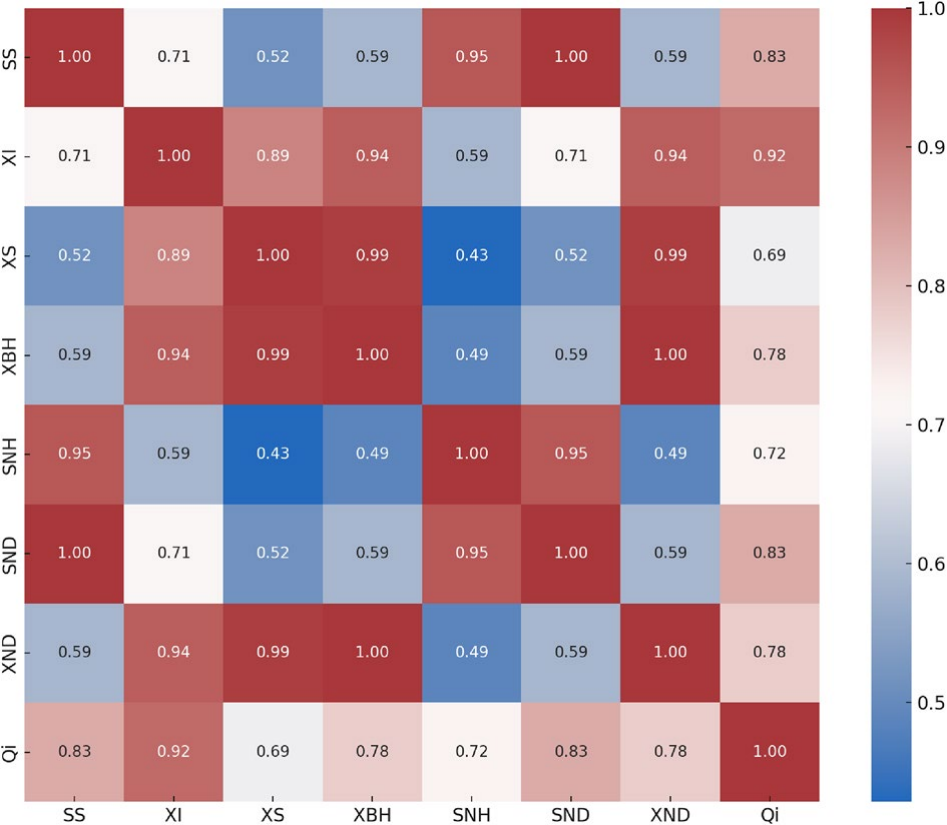
Correlation matrix of BSM1 influent variables under dry weather conditions.

The linear correlations among the variables are illustrated in Fig.  4 using a Pearson correlation heatmap.

The correlation analysis reveals several key relationships among the influent variables. A strong positive correlation is observed between $S_S$ and $X_S$, which is expected given their joint contribution to the overall chemical oxygen demand (COD) in the influent stream. Additionally, moderate correlations are evident between organic and nitrogen-related components, such as between $S_{ND}$ and $X_{ND}$, reflecting interconnected sources or transformation pathways in wastewater composition. In contrast, the flow rate $Q_i$ exhibits weak correlation with the other variables, consistent with its operational independence from the chemical characteristics of the influent. These correlation patterns support the application of ICA, as ICA is well-suited for separating mixed signals into statistically independent components, thereby improving the ability to isolate fault-related patterns that may be masked in correlated or redundant multivariate sensor data.

**Fig. 5:**
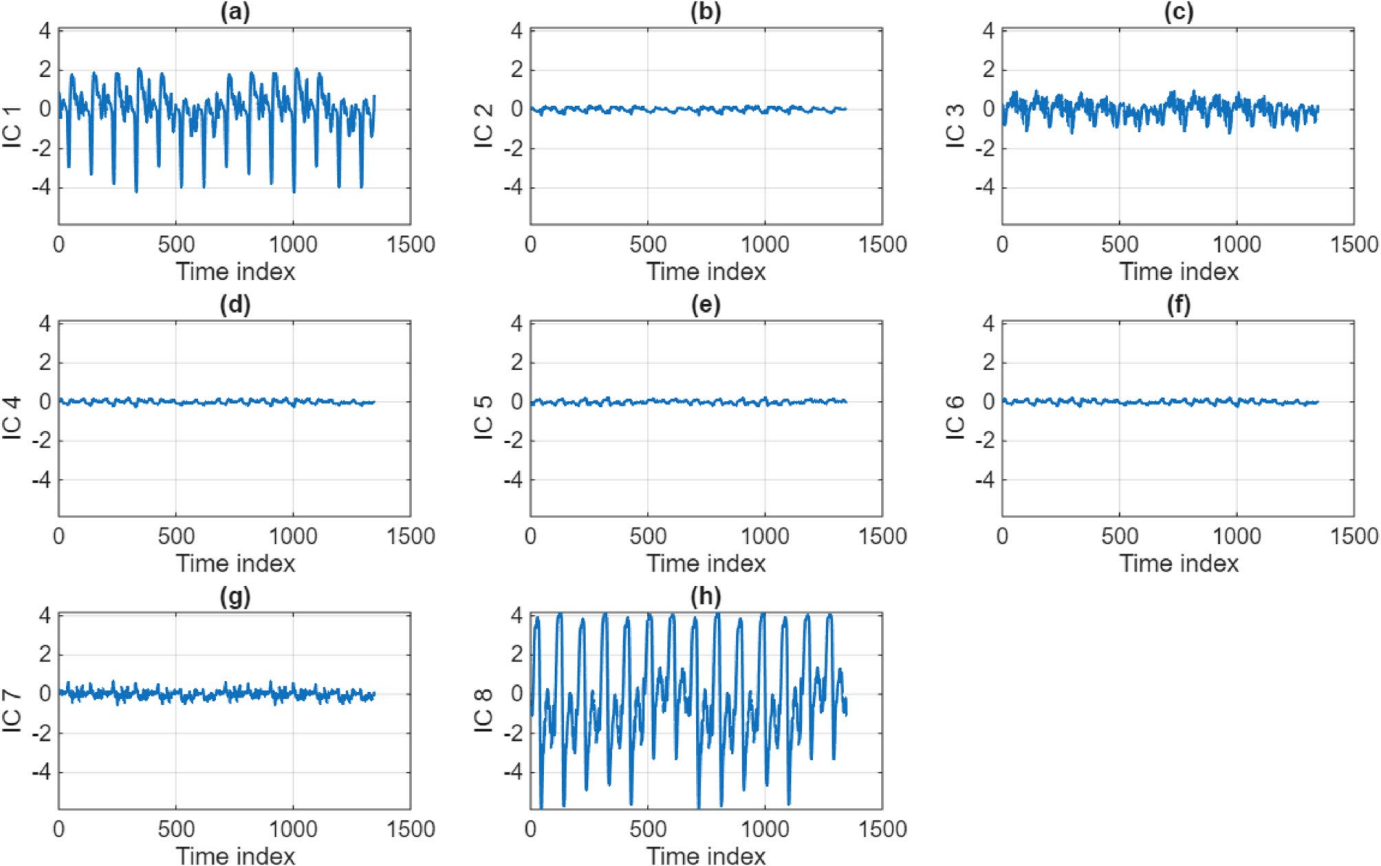
Time-series plots of the eight Independent Components (ICs) extracted via RICA: (**a**–**h**) show IC1–IC8, respectively. Each subplot shares the same y-axis range for consistent visual comparison across components.

Figure 5 presents the temporal evolution of the eight ICs obtained using Reconstruction ICA (RICA) applied to the BSM1 influent dataset. Notably, IC1 and IC8 exhibit strong, structured oscillatory patterns with higher variance, suggesting that they capture dominant modes of variation, possibly periodic behavior embedded in the original variables. In contrast, ICs 2 through 7 display low-amplitude fluctuations, with some appearing nearly constant or noise-like, indicating weaker contributions to the underlying process dynamics or representing residual noise. The shared y-axis range across subplots facilitates direct comparison, revealing the varying energy and informativeness of each component. These findings highlight the capability of RICA to isolate sparse, interpretable latent structures, which can be leveraged for efficient monitoring and anomaly detection.

**Fig. 6:**
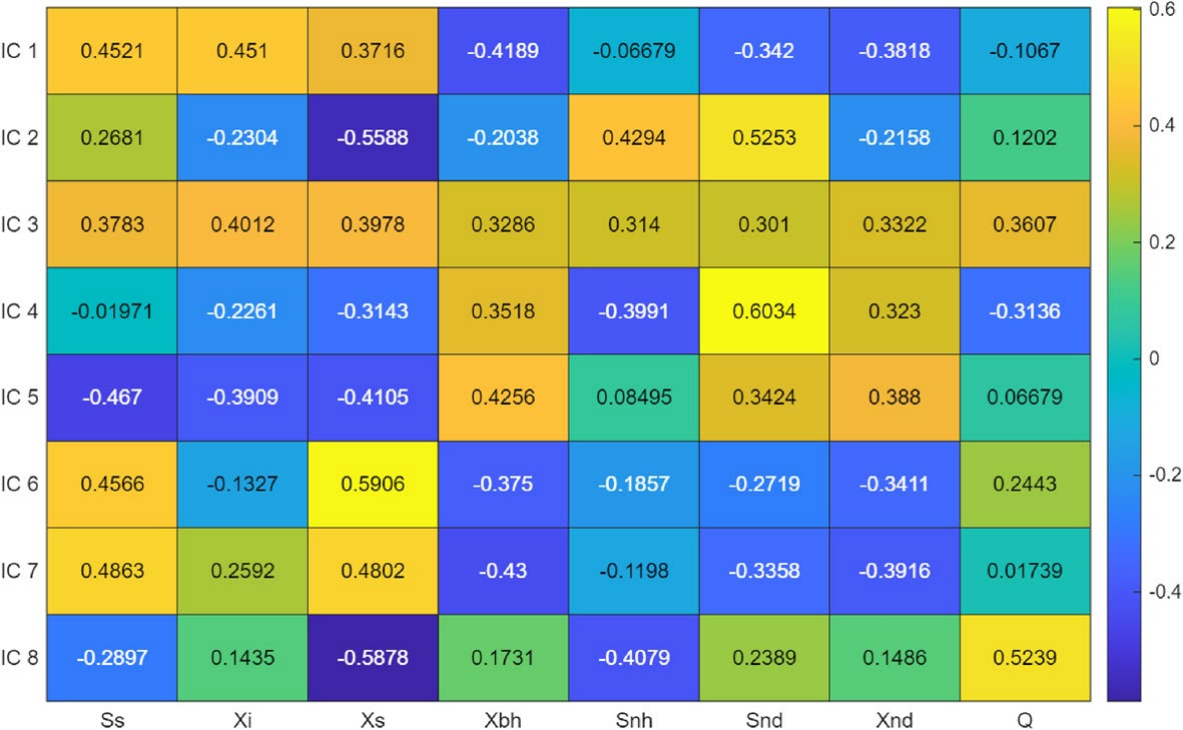
Mixing matrix obtained from RICA applied to the 8 selected influent variables of the BSM1 dataset. Each cell shows the weight of the original variable in the corresponding independent component (IC).

The mixing matrix obtained from the RICA (Reconstruction Independent Component Analysis) model is illustrated in Fig.  6. This matrix visualizes the contribution of each original variable to the independent components (ICs), thereby providing insights into the latent sources influencing the wastewater influent dynamics. Each row corresponds to an independent component, and each column represents one of the original variables. Higher absolute values indicate stronger influence of the corresponding variable on the IC.

Notably, ICs 1 and 3 exhibit high positive weights across multiple variables (Ss, Xi, Xs), suggesting they represent dominant common modes of variation in the organic substrate and inert matter. IC 2, on the other hand, is primarily influenced by Xs, Snd, and Snh, capturing dynamics specific to slowly biodegradable substrates and nitrogenous compounds. ICs 4 and 5 show a combination of positive and negative weights, with IC 4 strongly associated with Snd (0.6034) and negatively with Xnd, implying its potential relevance for monitoring organic nitrogen variability. IC 6 is predominantly driven by Xs (0.5906), while IC 8 is characterized by high positive influence from Q (0.5239) and negative loading from Xs (−0.5878), indicating a possible separation of hydraulic disturbances. Overall, this decomposition supports the assumption of underlying statistically independent sources influencing the process and highlights the separability of flow and compositional dynamics. Such interpretability is valuable for selecting relevant ICs in downstream anomaly detection, especially when aiming to isolate specific fault types or disturbances in the influent stream.

**Fig. 7:**
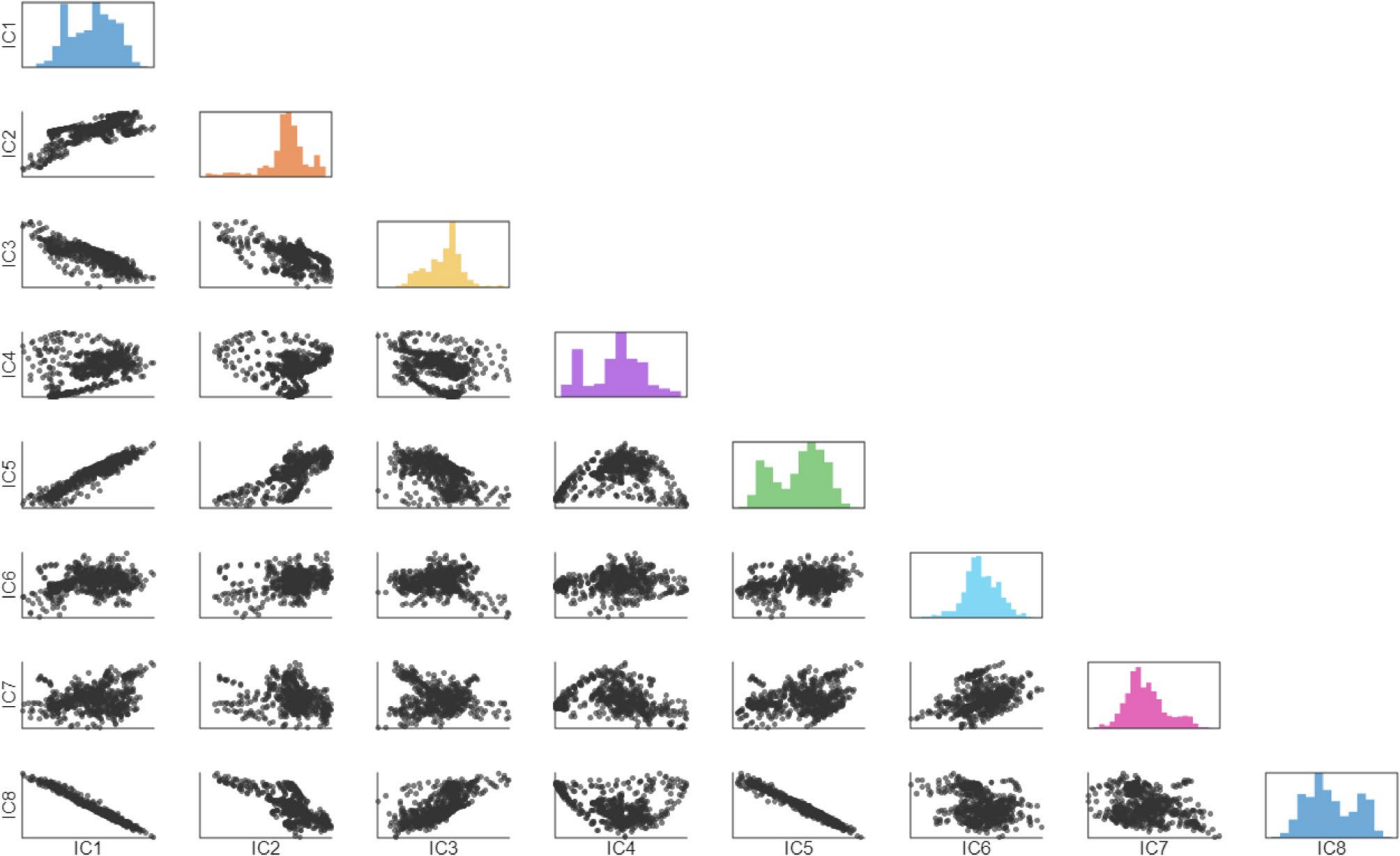
Lower-triangle pairwise scatter plots with diagonal histograms of the Independent Components obtained via RICA.

To evaluate the statistical independence and structural diversity of the extracted signals, we performed an ICA using the RICA algorithm on the standardized multivariate process dataset. The resulting independent components (ICs) were analyzed through a lower-triangle scatter plot matrix augmented with histograms along the diagonal (Fig.  7). This graphical layout provides insights into both the marginal distributions of each component and their pairwise dependencies. The scatter plots reveal distinct geometric patterns, with several components displaying nonlinear, elliptical, or fan-shaped relationships, indicating the success of the ICA in isolating non-Gaussian, statistically independent sources. Notably, some components exhibit clear decorrelation and sparsity, validating the effectiveness of the ICA transform in separating latent signals from the mixed process variables. The diagonal histograms further highlight the diversity in marginal distributions, ranging from near-Gaussian to skewed or multimodal shapes, supporting the non-Gaussian assumptions of ICA. This visualization serves as a compact and informative tool to interpret the learned latent representations and confirms the capability of ICA to uncover independent structure in complex process data.

### Detection results

**Fig. 8:**
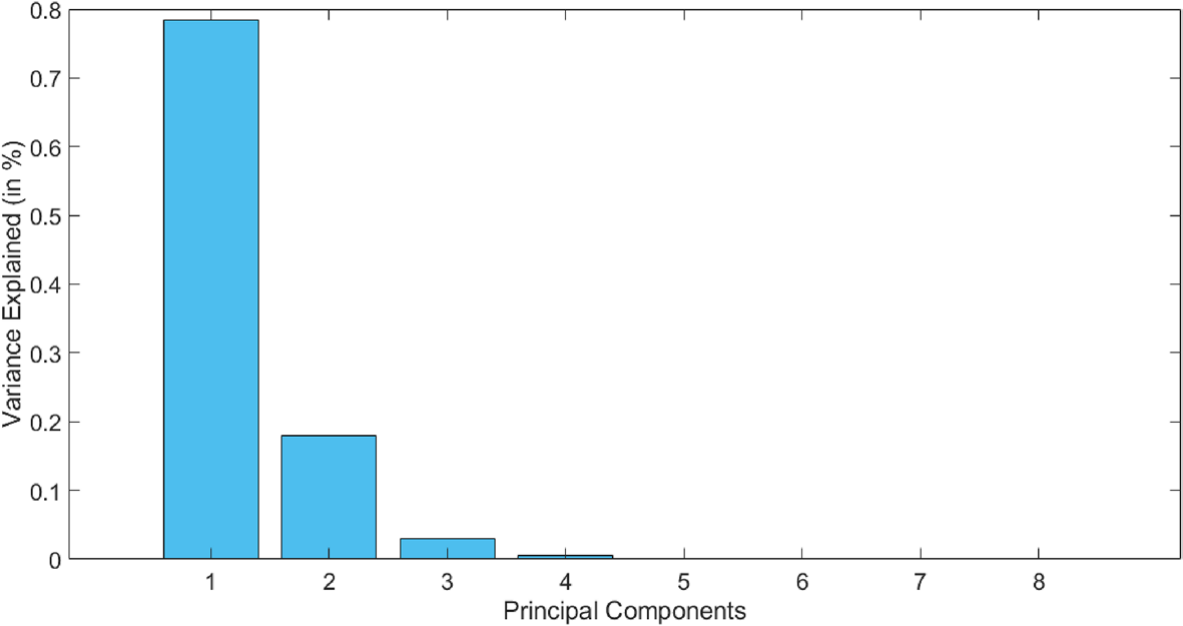
Cumulative variance explained by PCs for PCA strategy.

**Fig. 9:**
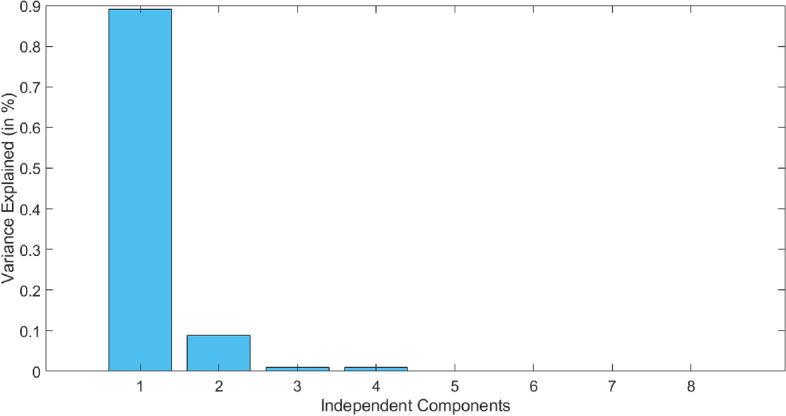
Cumulative variance explained by ICs for ICA strategy.

The dataset consists of 1,340 observations across eight influent variables, equally divided into training and testing subsets. Both PCA and ICA models were constructed using the fault-free training data. Selecting the number of independent components (ICs) is a critical step in ICA-based monitoring, as it directly affects the model’s ability to represent the underlying process behavior. If too few ICs are retained, important process information may be lost, reducing the method’s sensitivity and leading to missed detections, especially for subtle faults. On the other hand, retaining too many ICs may introduce noise-dominated components, which can degrade detection performance by increasing variability in the monitoring statistic and potentially leading to false alarms. In this study, the number of ICs was selected based on a CPV threshold of 95%, which is commonly recommended in the literature (typically between 80% and 95%). As shown in Fig.  8, three principal components (PCs) were selected for the PCA model using CPV approach, capturing the majority of the variance.As per the Fig.  9 three ICs are sufficient to capture the majority of the data variability, while the remaining components contribute marginally and are mainly associated with noise. Therefore, retaining three ICs ensures a good trade-off between detection sensitivity and robustness.

To enable dynamic monitoring, a moving window of size 30 samples was applied for computing the KS statistic, which quantifies distributional shifts in the residuals over time. The proposed ICA-KS-KDE fault detection (FD) strategy was evaluated under four representative fault scenarios injected into the $S_{NH}$ (ammonium) variable of the testing dataset. The considered fault scenarios are as follows:

- *Bias fault*: Introduced from time step 320 until the end of the testing set, with step magnitudes of 5%, 10%, 15%, and 20% of the variable’s dynamic range.
- *Intermittent fault*: A 5% variation fault injected over two time intervals: [125, 250] and [450, 575].
- *Drift fault*: A gradually increasing fault with a slope of 0.5, introduced from time step 350 to the end of the test sequence.
- *Freeze fault*: The sensor value was held constant at 6.0 from time step 320 onward, simulating a frozen sensor condition.

These fault scenarios represent diverse types of anomalies commonly encountered in wastewater treatment processes, including abrupt shifts, intermittent deviations, slow drifts, and signal loss resulting from sensor failure. The robustness of the ICA-KS approach under such diverse conditions demonstrates its potential as a general-purpose monitoring framework.

### Monitoring of bias faults with varying magnitudes

**Fig. 10:**
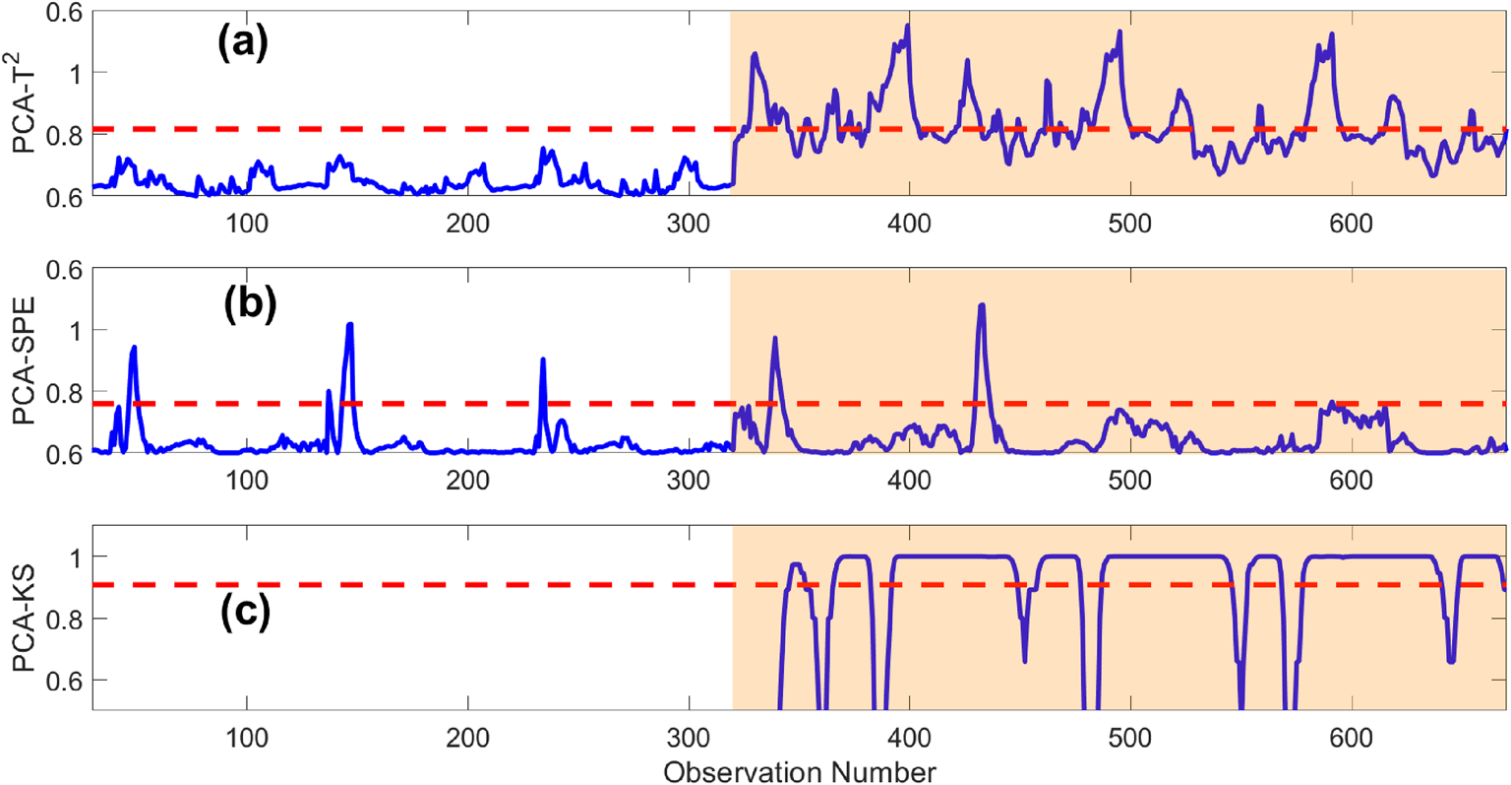
The performance of PCA detection methods illustrated for monitoring of bias fault: (**a**) PCA-$T^{2}$, (**b**) PCA-SPE, (**c**) PCA-KS.

**Fig. 11:**
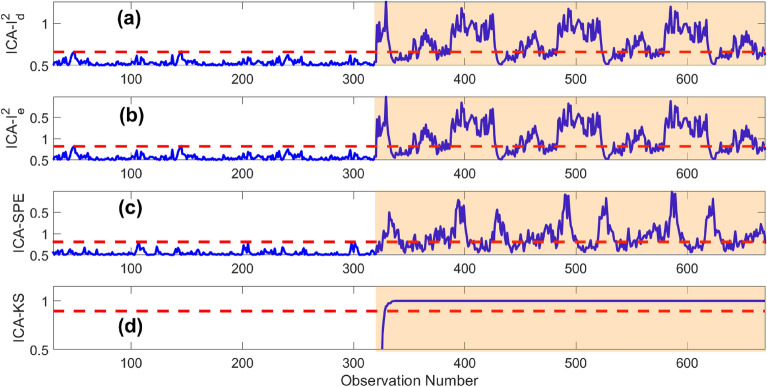
The performance of ICA detection methods illustrated for monitoring of bias fault: (**a**) ICA-$I^{2}_{d}$, (**b**) ICA-$I^{2}_{e}$, (**c**) ICA-SPE, (**d**) ICA-KS

Bias faults, characterized by a consistent offset in sensor readings, pose a critical threat to the reliable operation of WWTPs. Even moderate biases can lead to significant misinterpretations of process conditions, resulting in suboptimal control actions, increased energy consumption, and regulatory violations due to incorrect effluent quality estimations. Therefore, robust early detection of bias faults is essential for maintaining process safety and compliance. In this section, we evaluate the performance of the proposed ICA-KS-KDE strategy in detecting bias faults of varying magnitudes: 5%, 10%, 15%, and 20% of the total variation in the $S_{NH}$ variable. We focus first on the representative case of a 15% bias fault, with a comparative analysis of conventional PCA and ICA-based monitoring approaches. Figure 10 displays the detection results using three PCA-based indicators: $T^{2}$, SPE, and PCA-KS. The PCA-SPE index fails to detect the bias fault entirely, likely due to its sensitivity being limited to residual subspace variations rather than systematic drifts. While PCA-$T^{2}$ and PCA-KS exhibit partial detection, they still miss several faulty regions, suggesting inadequate sensitivity to small-to-moderate bias shifts, especially when fault signatures are entangled across multiple principal components. Conversely, the ICA-based fault indicators, shown in Fig.  11, demonstrate improved performance due to their ability to extract statistically independent components from non-Gaussian data. The standard ICA-$I^2_d$ and ICA-$I^2_e$ indicators capture a greater portion of the bias fault region, outperforming their PCA counterparts. However, minor gaps in detection persist, particularly at fault onset or under smaller deviations, indicating that ICA alone may not fully disentangle subtle shifts in the data distribution.

The proposed ICA-KS-KDE approach achieves superior performance, successfully detecting the entire bias fault period without missed detections. By leveraging the distributional sensitivity of the KS test and the data-adaptive thresholding capability of KDE, this hybrid strategy effectively identifies even low-magnitude biases. The combination of ICA residuals and non-parametric change detection enhances robustness against non-Gaussian noise and signal overlap, making it particularly suitable for real-time WWTP monitoring where early detection of systematic sensor faults is paramount.

The monitoring results for bias faults of varying magnitudes (5%, 10%, 15%, and 20%) are summarized in Table 3. Across all fault levels, the proposed ICA-KS strategy consistently achieves the highest detection rates (ADR), F1-scores, and overall accuracy. Notably, for low-magnitude faults (5% and 10%), traditional methods such as PCA-$T^2$, PCA-SPE, PCA-KS, and ICA-based indicators struggle to detect the fault, resulting in poor F1-scores. The PCA-SPE method, in particular, fails to detect the bias in all cases. This highlights the difficulty of capturing gradual or subtle deviations using variance-based or residual-space statistics alone. In contrast, the ICA-KS approach, by leveraging distributional changes in residuals and adaptive KDE-based thresholding, provides excellent sensitivity to small shifts, achieving near-perfect F1-scores of 98.60% and 99.41% for 5% and 10% faults, respectively. For higher magnitudes (15% and 20%), most conventional strategies begin to detect the bias more effectively; however, minor missed detections persist, slightly reducing their performance. The ICA-KS strategy, again, demonstrates superior robustness, attaining detection accuracies of 99.45% and 99.88%, confirming its reliability even under larger fault conditions. These results affirm the necessity of using a distribution-sensitive and data-adaptive approach for robust bias fault detection in WWTP monitoring.

**Table 3:** Comparison of fault detection performance under bias faults of varying magnitudes using different PCA- and ICA-based monitoring strategies.

Fault magnitude	Index	PCA-$T^{2}$	PCA-SPE	PCA-KS	ICA-$I^{2}_{d}$	ICA-$I^{2}_{e}$	ICA-SPE	ICA-KS
5*5%	ADR	44.74	6.05	62.12	48.77	51.45	53.22	97.25
	FAR	0.00	2.99	0.00	0.00	0.00	0.00	0.00
	Precision	100.00	70.00	100.00	100.00	100.00	100.00	100.00
	Accuracy	71.13	48.57	80.21	73.23	74.64	75.56	98.56
	F1-score	61.82	11.05	76.64	65.56	67.94	69.46	98.60
5*10%	ADR	62.91	6.77	76.12	63.77	66.87	74.85	98.85
	FAR	0.00	2.89	0.00	0.00	0.00	0.00	0.00
	Precision	100.00	72.72	100.00	100.00	100.00	100.00	100.00
	Accuracy	80.62	50.29	87.52	87.90	82.70	86.86	99.37
	F1-score	77.26	11.04	86.44	77.87	80.14	85.61	99.41
5*15%	ADR	75.29	7.25	83.85	76.77	77.54	81.15	99.25
	FAR	0.00	2.75	0.00	0.00	0.00	0.00	0.00
	Precision	100.00	74.28	100.00	100.00	100.00	100.00	100.00
	Accuracy	87.09	50.29	91.50	87.90	88.21	90.14	99.45
	F1-score	85.90	13.29	91.21	86.85	87.08	89.59	99.62
5*20%	ADR	90.45	15.45	98.50	92.55	93.75	96.85	99.81
	FAR	0.00	1.45	0.00	0.00	0.00	0.00	0.00
	Precision	100.00	91.74	100.00	100.00	100.00	100.00	100.00
	Accuracy	95.01	62.46	99.19	96.11	96.73	98.35	99.88
	F1-score	94.98	26.74	99.24	96.13	96.77	98.39	99.89

### Monitoring of drift faults with varying slopes

This section evaluates the performance of the proposed ICA-KS-KDE fault detection strategy for identifying drift faults with three different slopes: 0.07 (small), 0.5 (medium), and 2.0 (large). Table 4 summarizes the detection performance across various metrics. For the smallest slope (0.07), conventional indicators struggle to detect the slow-varying fault, leading to degraded accuracy and F1-scores. Notably, PCA-SPE fails, while PCA-$T^2$ and PCA-KS show moderate detection performance. In contrast, the ICA-KS approach significantly outperforms all others, achieving an F1-score of 95.42% and an accuracy of 98.75%. This demonstrates its strength in detecting gradual distributional shifts, which are often missed by variance- or residual-based methods. For the medium (0.5) and large (2.0) slopes, traditional indicators, including PCA-$T^2$, ICA-$I^2_d$, and ICA-SPE, begin to detect faults more reliably. However, the ICA-KS method not only improves detection accuracy (up to 99.87%) but also achieves earlier fault detection, as it captures subtle temporal trends through the KS-based distributional comparison. This advantage translates into higher F1-scores of 96.84% and 98.35%, confirming the robustness and precision of the proposed method across all drift fault scenarios.

**Table 4:** Detection performance of various PCA- and ICA-based strategies under drift faults with different slopes.

Slope	Index	PCA-$T^{2}$	PCA-SPE	PCA-KS	ICA-$I^{2}_{d}$	ICA-$I^{2}_{e}$	ICA-SPE	ICA-KS
5* **0.07**	ADR	61.77	11.42	70.55	64.27	64.88	69.35	91.25
	FAR	0.00	1.87	0.00	0.00	0.00	0.00	0.00
	Precision	100.00	94.78	100.00	100.00	100.00	100.00	100.00
	Accuracy	82.62	50.32	89.53	84.11	85.75	90.38	98.75
	F1-score	76.36	21.70	82.74	78.25	78.70	81.91	95.42
5*0.5	ADR	81.75	15.55	89.45	84.55	85.11	87.77	93.88
	FAR	0.00	3.22	0.00	0.00	0.00	0.00	0.00
	Precision	100.00	84.61	100.00	100.00	100.00	100.00	100.00
	Accuracy	89.83	54.32	97.27	95.11	95.67	97.13	99.51
	F1-score	81.51	26.31	94.42	91.62	91.95	93.48	96.84
5*2	ADR	86.54	25.12	93.78	89.31	90.76	91.73	96.75
	FAR	0.00	1.88	0.00	0.00	0.00	0.00	0.00
	Precision	100.00	94.92	100.00	100.00	100.00	100.00	100.00
	Accuracy	96.26	66.88	99.16	97.15	97.95	98.98	99.87
	F1-score	92.78	40.18	96.77	94.35	95.15	95.68	98.35

**Fig. 12:**
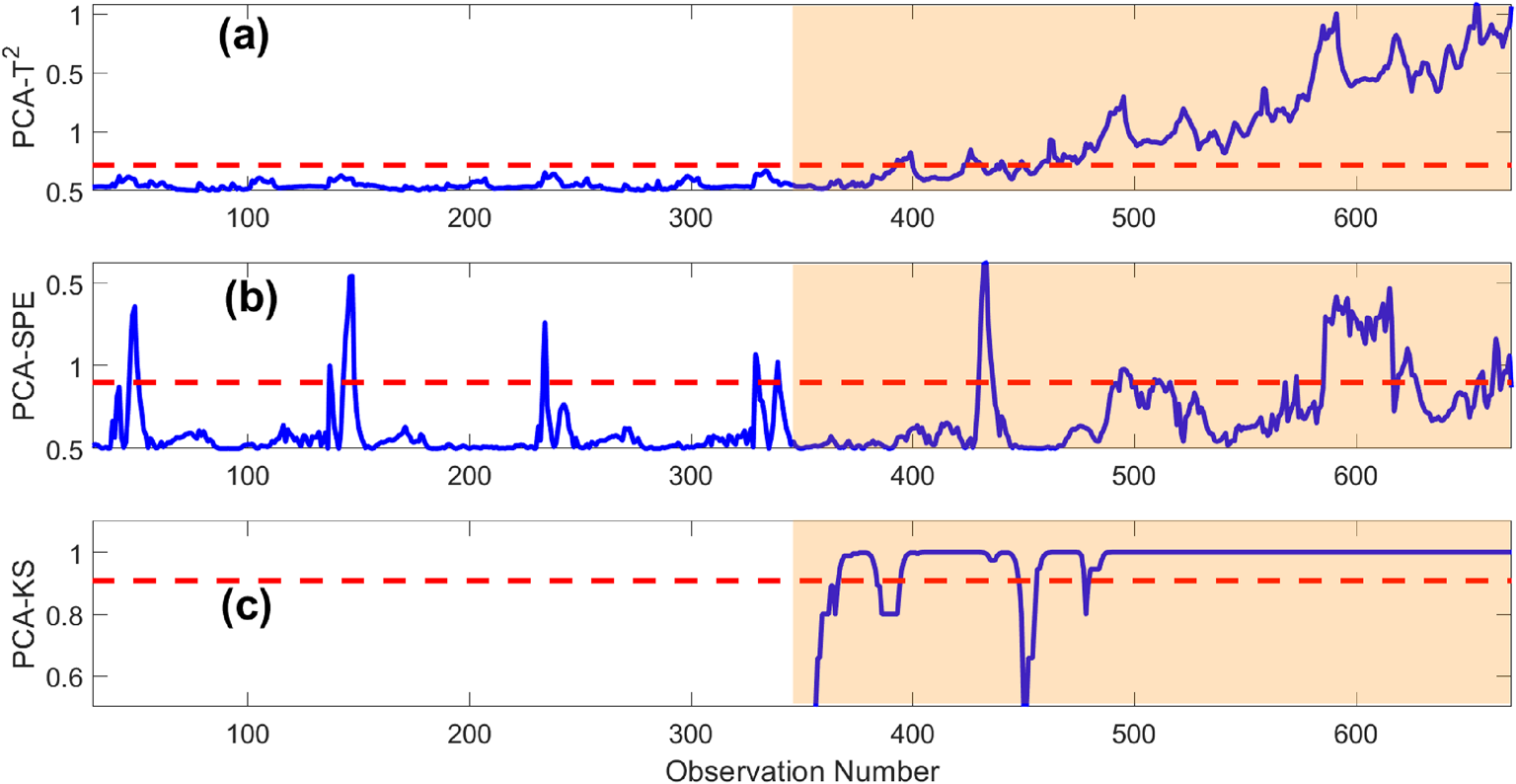
Monitoring of drift fault with slope = 0.5 using PCA-based indicators: (**a**) PCA-$T^2$, (**b**) PCA-SPE, (**c**) PCA-KS.

**Fig. 13:**
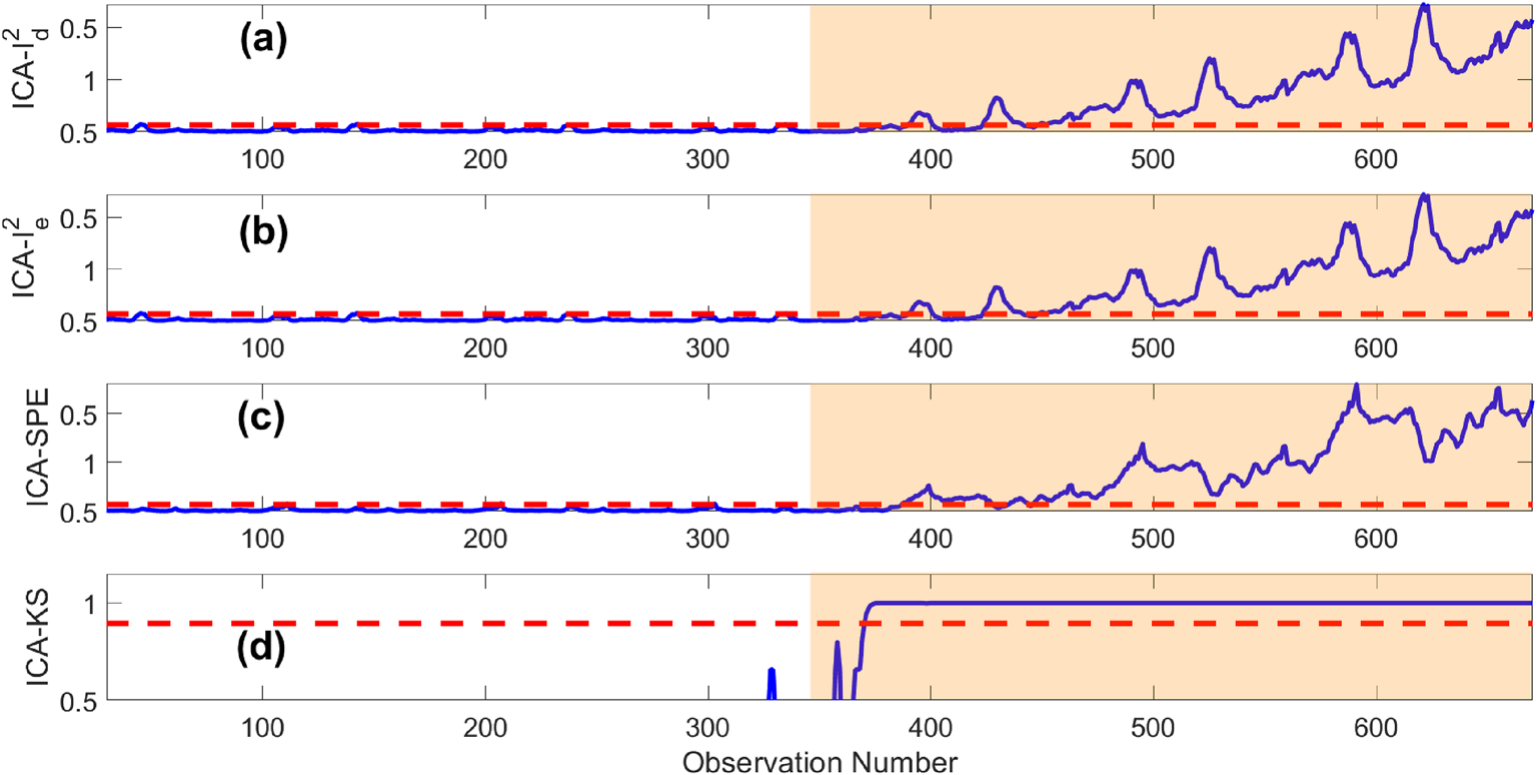
Monitoring of drift fault with slope = 0.5 using ICA-based indicators: (**a**) ICA-$I^2_d$, (**b**) ICA-$I^2_e$, (**c**) ICA-SPE, (**d**) ICA-KS.

To illustrate these findings, Figs.  12 and 13 present the detection results for a drift fault with a slope of 0.5. As shown in Fig.  12, the PCA-SPE method fails to detect the fault, while PCA-$T^2$ and PCA-KS offer partial detection with several missed regions. Figure 13 demonstrates that all ICA-based indicators improve detection performance, but only the ICA-KS method delivers consistent and precise fault detection throughout the fault duration.

### Monitoring of freezing fault

**Fig. 14:**
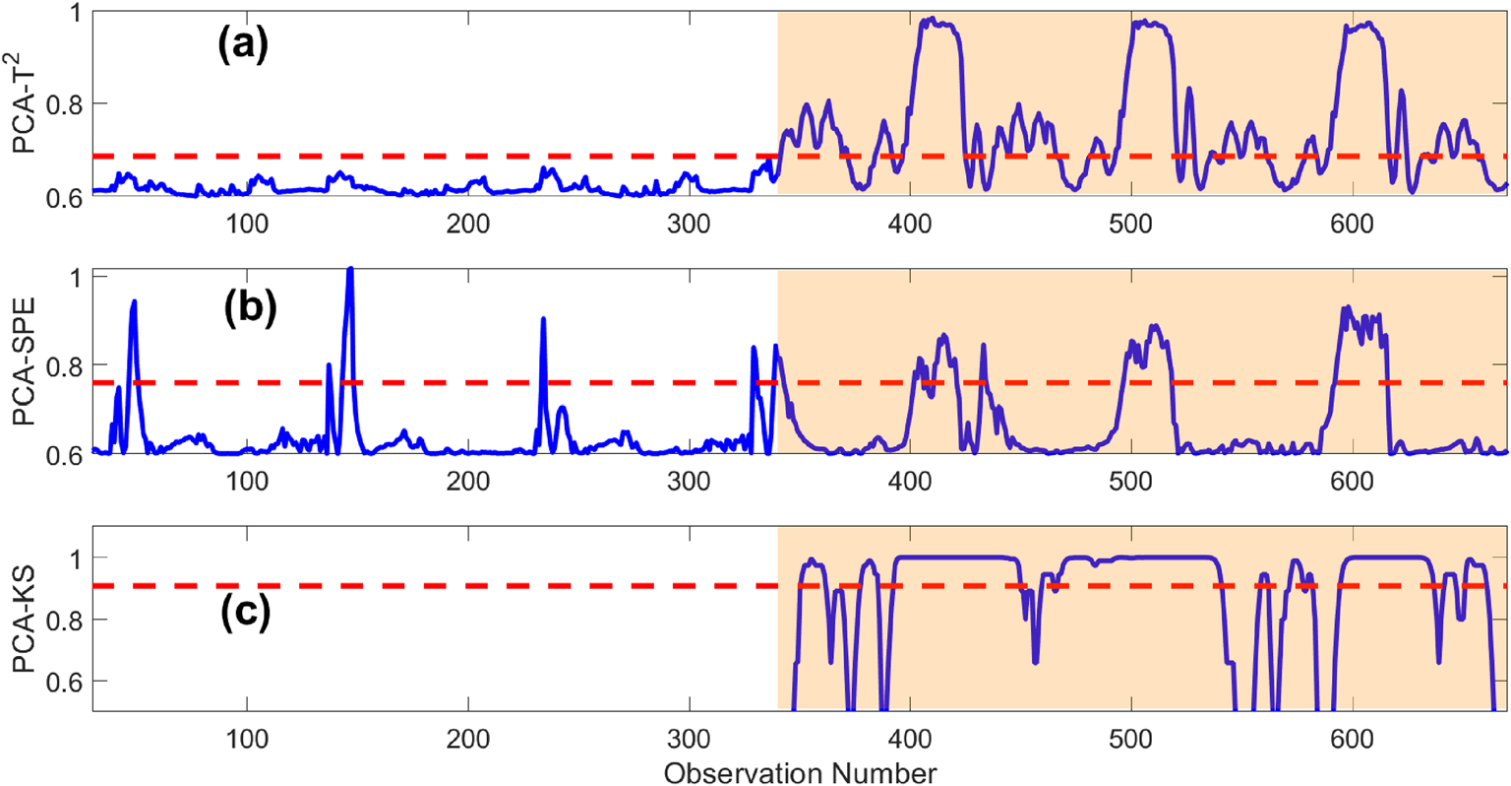
Monitoring of freezing fault using PCA-based indicators: (**a**) PCA-$T^2$, (**b**) PCA-SPE, (**c**) PCA-KS.

**Fig. 15:**
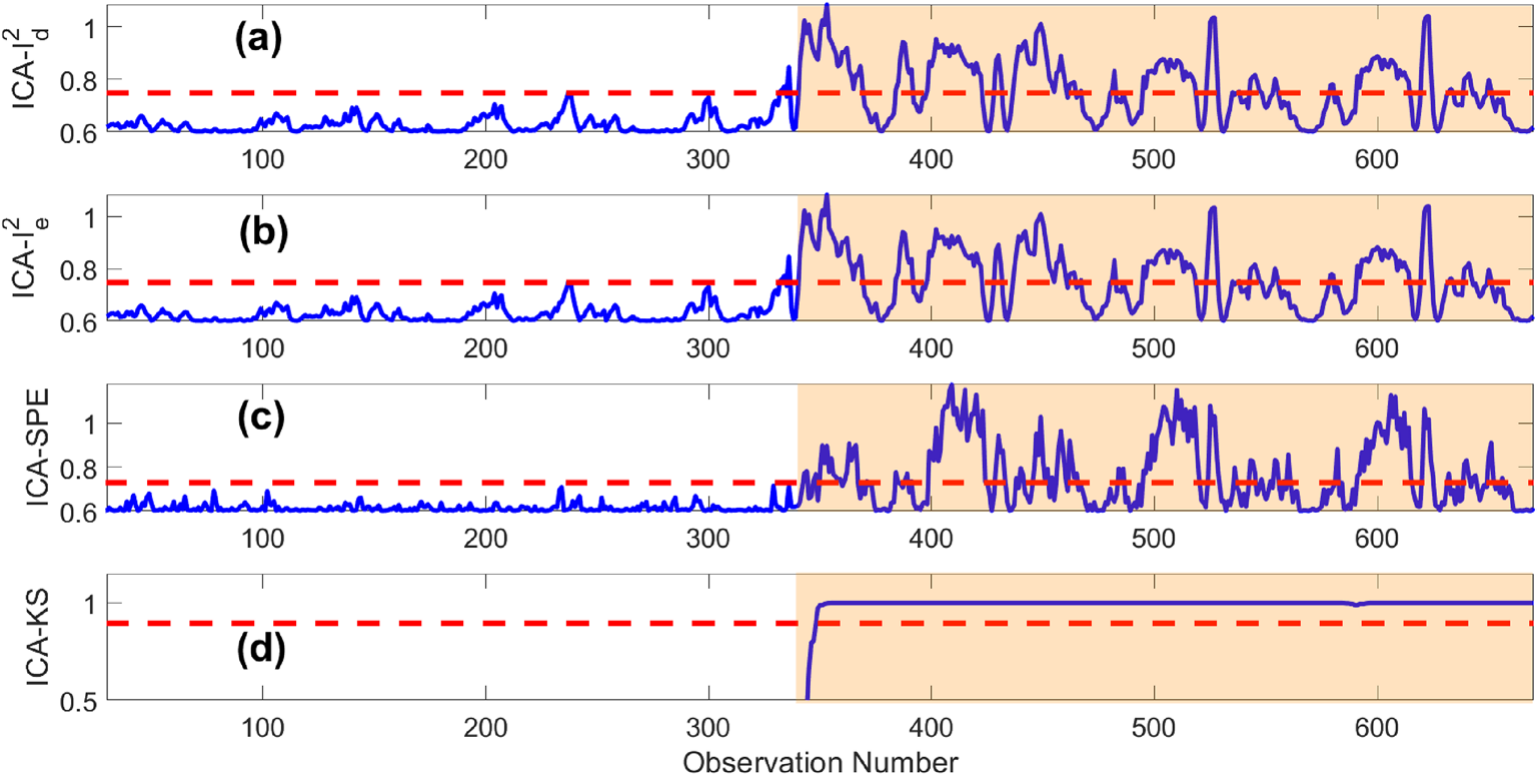
Monitoring of freezing fault using ICA-based indicators: (**a**) ICA-$I^2_d$, (**b**) ICA-$I^2_e$, (**c**) ICA-SPE, (**d**) ICA-KS.

This section evaluates the detection performance of PCA- and ICA-based strategies in identifying freezing faults. Figure 14 presents the results of the PCA-based indicators. It is evident that all three, PCA-$T^2$, PCA-SPE, and PCA-KS, fail to reliably detect the freezing fault. Among them, PCA-SPE completely fails, while PCA-$T^2$ and PCA-KS exhibit only limited sensitivity, with significant portions of the fault region undetected. Figure 15 displays the performance of the ICA-based methods. Conventional ICA indicators exhibit partial detection capabilities, but they fail to capture important portions of the faulty interval. In contrast, the proposed ICA-KS approach demonstrates robust and precise detection, with no missed detections and a consistent response once the fault occurs.

Table 5 summarizes the detection metrics for all approaches. The ICA-KS method clearly outperforms all others, achieving an average detection rate (ADR) of 97.66%, an accuracy of 98.77%, and an F1-score of 98.82%. These results highlight the importance of distribution-based monitoring in detecting abrupt and sustained sensor freezing events. Traditional PCA- and ICA-based variance- or residual-driven strategies exhibit weak sensitivity to such abrupt yet static shifts, underscoring the need for adaptive and nonparametric indicators, such as KS-based monitoring.

**Table 5:** Detection performance of PCA- and ICA-based strategies under freezing fault scenario.

Index	PCA-$T^{2}$	PCA-SPE	PCA-KS	ICA-$I^{2}_{d}$	ICA-$I^{2}_{e}$	ICA-SPE	ICA-KS
ADR	63.71	21.71	71.75	68.85	80.45	69.76	97.66
FAR	0.00	3.15	0.00	0.00	0.00	0.00	0.00
Precision	100.00	88.37	100.00	100.00	100.00	100.00	100.00
Accuracy	81.05	58.51	85.24	83.24	89.78	84.21	98.77
F1-score	77.84	34.88	83.55	81.55	89.15	82.18	98.82

### Monitoring of intermittent faults with varying magnitudes

This section evaluates the performance of PCA- and ICA-based monitoring strategies under intermittent faults of varying magnitudes: 5%, 10%, 15%, and 20%, injected into the variable $S_{NH}$ at intervals [125, 250] and . Table 6 summarizes the detection metrics across all methods. For high (15%) and very high (20%) magnitudes, most methods, except PCA-SPE, achieve satisfactory detection, as the fault behaves like a strong bias, making it easier to detect. However, even in these scenarios, ICA-KS surpasses others, achieving F1-scores of 99.35% and 99.65% with zero false alarms.

**Fig. 16:**
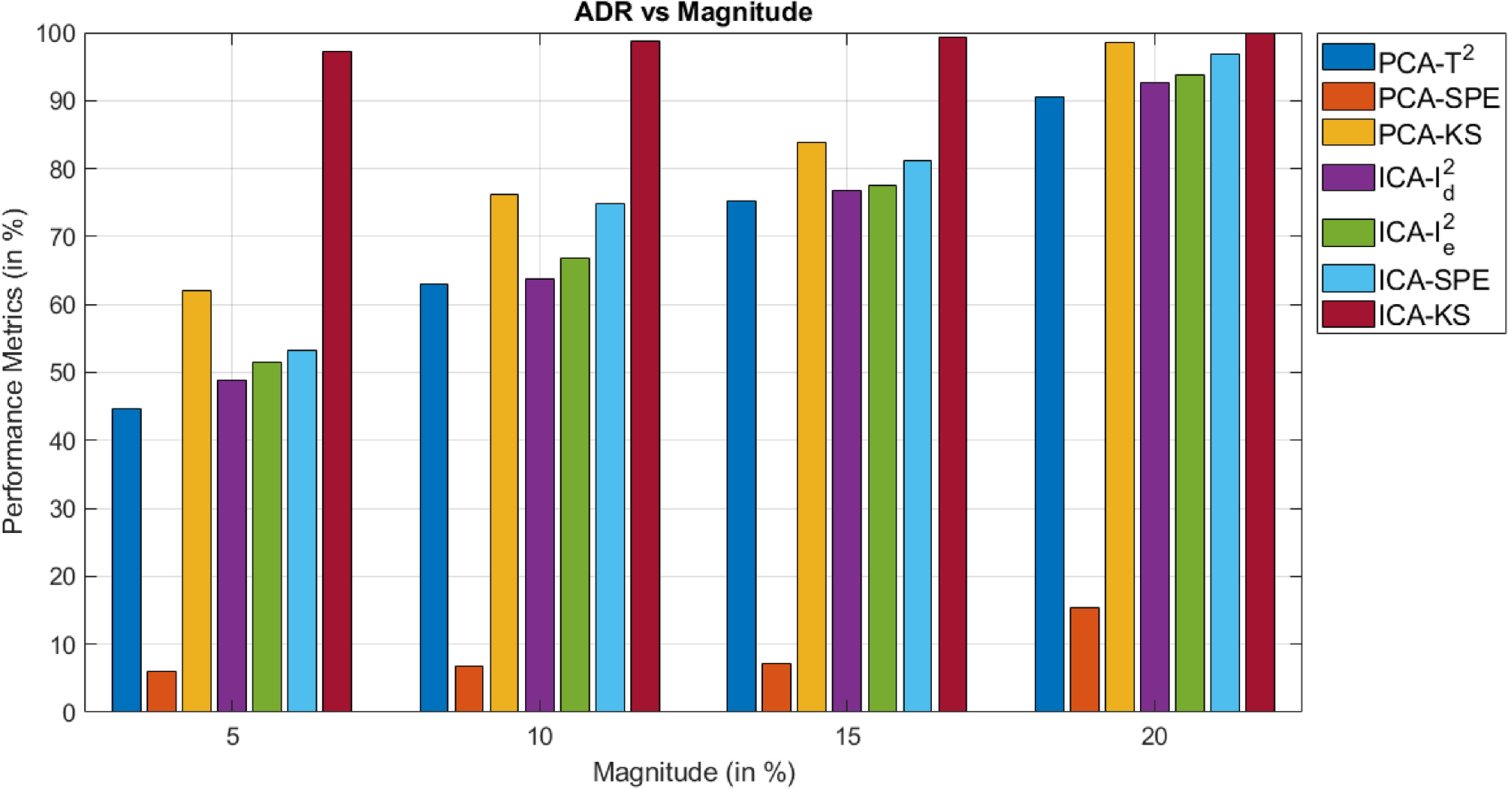
Variation of average detection rate (ADR) with intermittent fault magnitude.

**Fig. 17:**
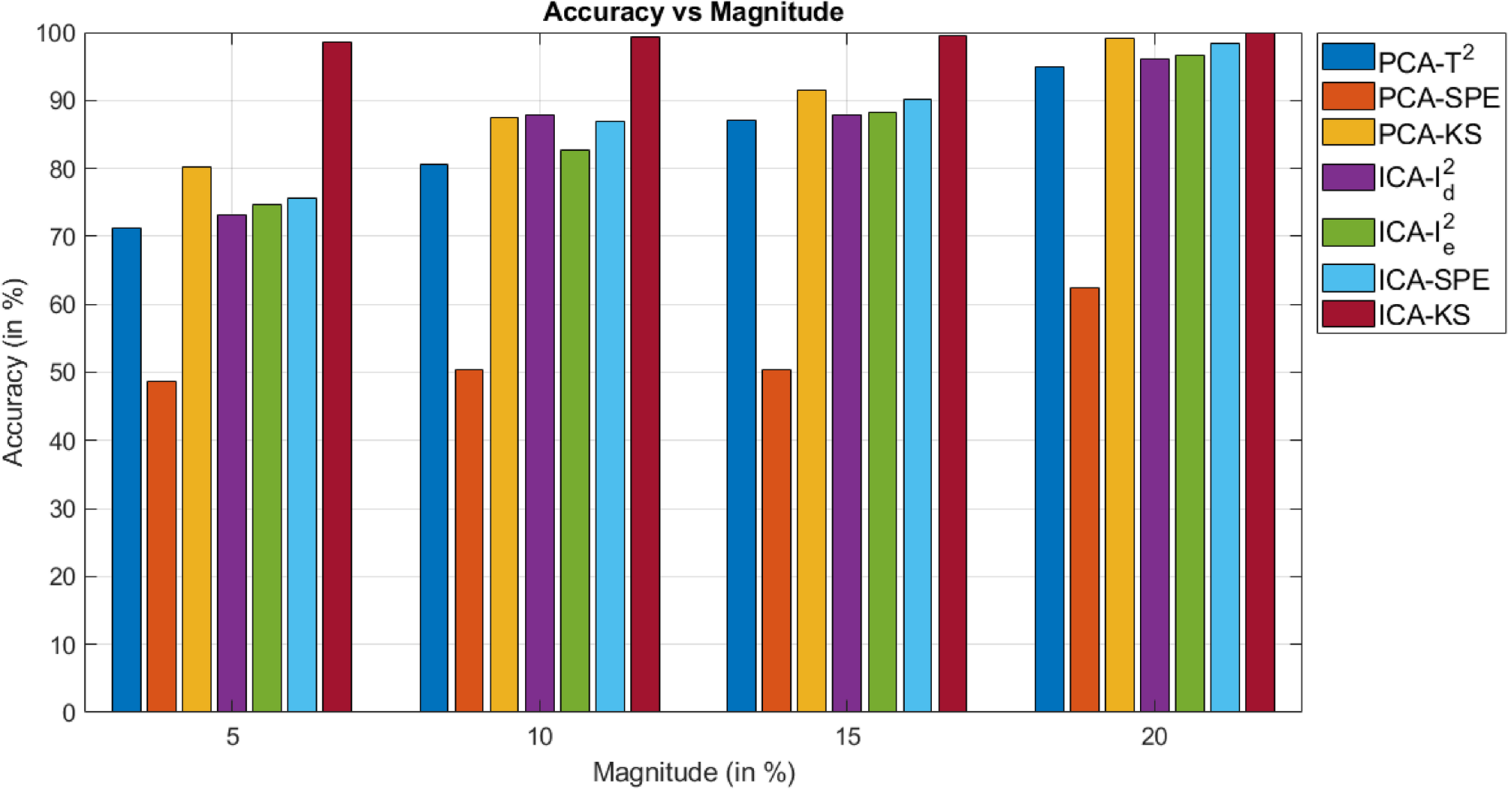
Variation of accuracy with intermittent fault magnitude.

For medium (10%) and especially small (5%) fault magnitudes, all conventional PCA and ICA indicators exhibit degraded performance, with F1-scores falling as low as 8–72%, reflecting their insensitivity to subtle, intermittent deviations. In stark contrast, ICA-KS remains highly effective, achieving 98.96% and 98.53% F1-scores, respectively. This superior performance is attributed to its ability to detect localized, sample-wise distributional changes, which conventional variance-based or residual-driven indicators often fail to capture. Figs. 16 and 17 illustrate the consistent dominance of ICA-KS in terms of ADR and accuracy across all fault magnitudes.

**Table 6:** Detection performance under intermittent faults of varying magnitudes.

Fault magnitude	Index	PCA-$T^{2}$	PCA-SPE	PCA-KS	ICA-$I^{2}_{d}$	ICA-$I^{2}_{e}$	ICA-SPE	ICA-KS
5* **5%**	ADR	41.32	4.05	55.12	43.64	45.88	55.92	97.11
	FAR	0.00	0.00	0.00	0.00	0.00	0.00	0.00
	Precision	100.00	100.00	100.00	100.00	100.00	100.00	100.00
	Accuracy	68.55	47.66	77.53	69.25	71.05	79.88	98.28
	F1-score	58.47	8.58	71.06	60.76	62.90	71.72	98.53
5*10%	ADR	57.44	4.92	65.38	58.91	55.45	72.64	97.95
	FAR	0.00	0.00	0.00	0.00	0.00	0.00	0.00
	Precision	100.00	100.00	100.00	100.00	100.00	100.00	100.00
	Accuracy	77.33	49.75	83.44	83.43	78.52	85.77	99.00
	F1-score	72.96	9.61	79.06	74.11	71.34	84.16	98.96
5*15%	ADR	68.77	5.77	71.45	69.31	69.55	85.88	98.75
	FAR	0.00	0.00	0.00	0.00	0.00	0.00	0.00
	Precision	100.00	100.00	100.00	100.00	100.00	100.00	100.00
	Accuracy	83.73	50.89	85.07	84.02	84.17	92.53	99.25
	F1-score	81.48	11.42	83.34	81.87	82.33	92.42	99.35
5* **20%**	ADR	84.25	13.54	92.37	84.15	86.22	91.66	99.25
	FAR	0.00	0.00	0.00	0.00	0.00	0.00	0.00
	Precision	100.00	91.74	100.00	100.00	100.00	100.00	100.00
	Accuracy	93.15	60.05	95.65	93.45	94.12	95.88	99.75
	F1-score	91.45	24.02	96.03	91.82	93.20	95.65	99.65

### Sensitivity analysis for different window sizes

In this section, sensitivity analysis is performed for three different window sizes, namely, 20, 30 and 40 respectively using FDR and FAR based statistical indicies. The performance of PCA-KS and ICA-KS based strategies is assessed for different window sizes while monitoring bias, freeze and drift faults respectively. The results are reported in Table 7. It is observed that a smaller window size ($w=20$) results in lower detection rates due to insufficient statistical representation, whereas a larger window size ($w=40$) introduces smoothing effects that delay fault detection. In contrast, $w=30$ provides the best trade-off, achieving the highest detection rates without delay. Notably, the false alarm rate (FAR) remains zero across all configurations. Therefore, a window size of 30 is selected as it ensures optimal detection performance and timely fault identification.

**Table 7:** Comparison of FDR and FAR for different window sizes.

	Method	Size=20		Size=30		Size=40	
		FDR	FAR	FDR	FAR	FDR	FAR
Bias	PCA-KS	59.85	0.00	62.12	0.00	61.85	0.00
	ICA-KS	95.29	0.00	97.25	0.00	96.65	0.00
Freeze	PCA-KS	67.87	0.00	71.75	0.00	70.45	0.00
	ICA-KS	94.50	0.00	97.66	0.00	96.65	0.00
Drift	PCA-KS	85.01	0.00	89.45	0.00	88.41	0.0
	ICA-KS	90.91	0.00	93.88	0.00	92.85	0.00

### Monitoring of multiple simultaneous faults

This section evaluates the monitoring performance of different strategies when two faults occur simultaneously in different variables. Specifically, a freezing fault is introduced in variable $S_{NH}$, where the signal is fixed at 40 after time instant 320, and a bias fault (10% of total variation) is introduced in $Q_{i}$ at the same point in time. The simultaneous faults were selected to represent a realistic and challenging operational scenario in wastewater treatment plants. $S_{NH}$ reflects the efficiency of biological processes, particularly nitrification, and is prone to sensor freezing due to fouling or communication issues. In contrast, $Q_i$ governs influent loading conditions and may be biased due to flow meter miscalibration. These variables are physically coupled, as changes in flow directly affect ammonia dynamics. Introducing faults in both variables simultaneously creates a coupled disturbance scenario where fault effects overlap. This provides a stringent test of the proposed ICA-KS framework, demonstrating its ability to detect and separate mixed fault signatures under realistic multi-fault conditions.

Table 8 reports the detection metrics for all considered methods.

**Table 8:** Monitoring performance under two simultaneous faults.

Index	PCA-$T^{2}$	PCA-SPE	PCA-KS	ICA-$I^{2}_{d}$	ICA-$I^{2}_{e}$	ICA-SPE	ICA-kS
ADR	79.95	5.45	88.94	83.32	88.11	52.40	96.50
FAR	0.00	1.22	0.00	0.00	0.00	0.00	0.00
Precision	100.00	82.32	100.00	100.00	100.00	100.00	100.00
Accuracy	90.28	42.45	96.37	93.12	95.07	72.53	99.65
F1-score	88.85	5.47	94.14	90.90	93.68	68.75	98.26

Results show that PCA-SPE fails to detect the simultaneous faults, while PCA-$T^{2}$, PCA-KS, ICA-$I^{2}{d}$, ICA-$I^{2}{e}$, and ICA-SPE achieve only partial detection with missed alarms during the faulty period, yielding accuracies between 72.53% and 96.37% and F1-scores between 68.75% and 94.14%. In contrast, the proposed ICA-KS strategy delivers superior performance, achieving 99.65% accuracy and a 98.26% F1-score without false alarms, owing to the KS test’s sensitivity to sample-level distributional changes combined with ICA’s ability to isolate independent fault-related sources, even under concurrent fault conditions.

### Fault isolation for drift fault

**Fig. 18:**
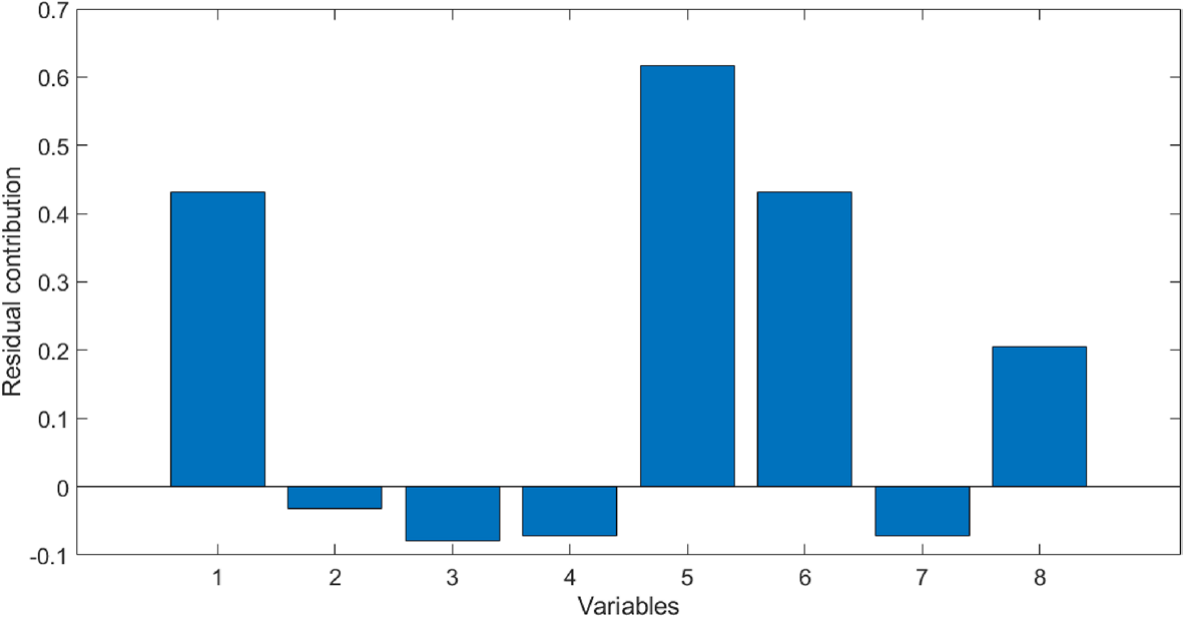
Contribution plot for a drift fault.

This section describes the fault isolation plot which has been utilized to determine the root cause of the fault. To illustrate , we present a contribution plot for a drift fault scenario. A drift fault with a slope of 0.5 was introduced in the $S_{NH}$ (ammonium) variable (variable 5) from time step 350 onwards until the end of the testing data. The contribution plot scenario for the drift fault is presented in Fig. 18. As shown in Fig. 18, the variable 5 exhibits the largest contribution compared to the other variables, clearly identifying it as the root cause of the fault.

### Emperical computation feasibility

In this section, the empirical execution times measured during the testing phase for all considered methods are discussed. The results are reported in Table 9. As shown, the computation time for all methods remains very low (on the order of milliseconds per sample), confirming their suitability for real-time implementation. The PCA-KS and ICA-KS methods involve moving-window computations, which introduce a slight additional overhead compared to conventional statistics. However, this increase remains negligible in practice. Importantly, the proposed ICA-KS method achieves significantly improved detection performance while maintaining low computational cost, demonstrating a favorable trade-off between accuracy and efficiency. These results support the real-time feasibility of the proposed framework for online monitoring applications.

**Table 9:** Execution time of different methods considered in this study.

Strategy	Time (seconds)
PCA-${\mathrm{T}}^{2}$	0.00514
PCA-SPE	0.00448
PCA-KS	0.00272
ICA-${\mathrm{I}}_{d}^{2}$	0.00553
ICA-${\mathrm{I}}^{2}_{e}$	0.00527
ICA-SPE	0.00467
ICA-KS	0.00274

Overall, the results across all tested fault scenarios, bias, drift, freezing, intermittent, and simultaneous faults, clearly highlight the superior robustness and sensitivity of the proposed ICA-KS strategy. While conventional PCA and ICA-based indicators often fail to detect weak or non-Gaussian faults or suffer from missed detections, ICA-KS consistently achieves near-perfect accuracy and F1-scores, even under challenging conditions such as small-magnitude or overlapping faults. Its strength lies in combining source separation with nonparametric, sample-wise distributional comparison, enabling reliable detection with minimal false alarms. This makes ICA-KS a strong candidate for deployment in real-time industrial monitoring applications where robustness, early detection, and generalization across fault types are critical.

## Conclusion

Faults in WWTPs can severely disrupt operational efficiency, compromise effluent quality, and incur significant financial losses. To address this critical challenge, we proposed a robust fault detection (FD) strategy that combines Independent Component Analysis (ICA) with the non-parametric Kolmogorov–Smirnov (KS) test and Kernel Density Estimation (KDE). This ICA–KS–KDE framework leverages ICA’s ability to extract statistically independent components from multivariate process data, while the KS test performs sample-by-sample comparison of empirical residual distributions across a moving window to detect subtle deviations from normal behavior. KDE was further used to compute adaptive thresholds, enhancing the sensitivity and generalizability of the method. Comprehensive evaluations were conducted on a simulated WWTP dataset that covered various realistic fault types, including bias, drift, freezing, and intermittent faults, under different magnitudes and slopes. The proposed ICA–KS strategy consistently outperformed conventional PCA- and ICA-based detection methods, demonstrating higher detection rates, accuracy, and F1-scores, particularly in challenging scenarios such as low-magnitude or slowly evolving faults. Moreover, even under simultaneous fault conditions affecting multiple variables, the ICA–KS method maintained robust performance without false alarms, highlighting its effectiveness in complex fault scenarios. The results confirm that the ICA–KS approach is a powerful and interpretable FD framework for real-time anomaly monitoring in industrial settings such as WWTPs. Its strength lies in its non-parametric nature, high sensitivity to subtle distributional changes, and capability to isolate meaningful process deviations even under concurrent fault conditions.

Future work will explore two key methodological directions. (1) Dynamic extensions of the ICA–KS framework, incorporating time-lagged features or Dynamic ICA to better capture temporal dependencies and evolving fault patterns. (2) Integration of explainable AI (XAI) modules, such as SHAP or counterfactual analysis, to improve the interpretability of fault causes and enable actionable decision support for plant operators. These advances aim to further improve detection accuracy, real-time responsiveness, and transparency in complex multivariate industrial systems. Further, the proposed framework demonstrates strong performance on benchmark datasets. Validation using real operational data will further enhance its practical relevance. Ongoing efforts are directed toward accessing real wastewater treatment plant datasets through collaborative studies, which will be considered an important direction for future work.

## Data Availability

The datasets generated during and/or analysed during the current study are available in Copp J B. The COST Simulation Benchmark: Description and Simulator Manual: Office for Official Publications of the European Community. Luxembourg: ISBN 92-894-1658-0; 2002. https://op.europa.eu/en/publication-detail/-/publication/8448ef88-37dd-4d1a-823f-3143e7902429/language-en
